# The Na^+^-activated K^+^ channel Slack contributes to synaptic development and plasticity

**DOI:** 10.1007/s00018-021-03953-0

**Published:** 2021-10-18

**Authors:** Lucas Matt, Thomas Pham, David Skrabak, Felix Hoffmann, Philipp Eckert, Jiaqi Yin, Miriam Gisevius, Rebekka Ehinger, Anne Bausch, Marius Ueffing, Karsten Boldt, Peter Ruth, Robert Lukowski

**Affiliations:** 1 Department of Pharmacology, Toxicology and Clinical Pharmacy, Institute of Pharmacy, University of Tübingen, 72076 Tübingen, Germany; 2 Institute for Ophthalmic Research, Molecular Biology of Retinal Degenerations and Medical Bioanalysis, University of Tübingen, 72076 Tübingen, Germany

**Keywords:** Na^+^-activated K^+^ channel, Slack, Synaptic plasticity, Intellectual disability, Long-term depression (LTD), NMDA receptor

## Abstract

Human mutations of the Na^+^-activated K^+^ channel Slack (*KCNT1*) are associated with epilepsy and intellectual disability. Accordingly, Slack knockout mice (*Slack*^*−/−*^) exhibit cognitive flexibility deficits in distinct behavioral tasks. So far, however, the underlying causes as well as the role of Slack in hippocampus-dependent memory functions remain enigmatic. We now report that infant (P6–P14) *Slack*^*−/−*^ lack both hippocampal LTD and LTP, likely due to impaired NMDA receptor (NMDAR) signaling. Postsynaptic GluN2B levels are reduced in infant *Slack*^*−/−*^, evidenced by lower amplitudes of NMDAR-meditated excitatory postsynaptic potentials. Low GluN2B affected NMDAR-mediated Ca^2+^-influx, rendering cultured hippocampal *Slack*^*−/−*^neurons highly insensitive to the GluN2B-specific inhibitor Ro 25-6981. Furthermore, dephosphorylation of the AMPA receptor (AMPAR) subunit GluA1 at S845, which is involved in AMPAR endocytosis during homeostatic and neuromodulator-regulated plasticity, is reduced after chemical LTD (cLTD) in infant *Slack*^*−/−*^. We additionally detect a lack of mGluR-induced LTD in infant *Slack*^*−/−*^, possibly caused by upregulation of the recycling endosome-associated small GTPase Rab4 which might accelerate AMPAR recycling from early endosomes. Interestingly, LTP and mGluR LTD, but not LTD and S845 dephosphorylation after cLTD are restored in adult *Slack*^*−/−*^. This together with normalized expression levels of GluN2B and Rab4 hints to developmental “restoration” of LTP expression despite Slack ablation, whereas in infant and adult brain, NMDAR-dependent LTD induction depends on this channel. Based on the present findings, NMDAR and vesicular transport might represent novel targets for the therapy of intellectual disability associated with Slack mutations. Consequently, careful modulation of hippocampal Slack activity should also improve learning abilities.

**Supplementary Information:**

The online version contains supplementary material available at 10.1007/s00018-021-03953-0.

## Introduction

Intellectual disability (ID) is a heterogeneous neurodevelopmental disorder affecting 1–3% of the population [1]. It is characterized by reduced intellectual (IQ < 70) and adaptive functioning [2]. Human mutations of *KCNT1*, the gene coding for the Na^+^-activated K^+^ channel Slack (Sequence Like a Ca^2+^-activated K^+^ Channel, K_Na_2.1, Slo2.2) cause at least two childhood epilepsy syndromes called malignant migrating focal seizures of infancy (MMFSI) and autosomal-dominant nocturnal frontal lobe epilepsy (ADNFLE) [3] associated with severe ID [4–6]. Interestingly, this inherited ID is not due to seizures but to altered Slack function, as it is not observed in patients suffering from identical epilepsy syndromes caused by unrelated mutations [6, 7]. With one exception [8, 9], all epilepsy-related mutations identified increased Slack activity. All of them, however, are tightly associated with high ID incidence [8–11].

Slack is broadly expressed in brain [5, 12–14], spinal cord and peripheral sensory neurons [15–17]. It contributes to neuronal resting membrane potential, action potential repolarisation and afterhyperpolarization as well as firing rate adaptation [18, 19].

While vitality and fertility of Slack knockout mice (*Slack*^*−/−*^) is normal, they display increased sensitivity to neuropathic pain [16], decreased cognitive flexibility [5] and impaired motor skill learning [20], indicating compromised plasticity of glutamatergic synapses in *Slack*^*−/−*^ [21]. Glutamate, the major neurotransmitter for fast excitatory neurotransmission [22] activates postsynaptic AMPA-type glutamate receptors (AMPAR). Specific synaptic activity patterns provoke postsynaptic Ca^2+^ influx through NMDA-type glutamate receptors (NMDAR). Glutamate release in response to presynaptic high frequency stimulation (HFS) generates large postsynaptic increases in intracellular Ca^2+^ which strengthen the synapse through integration of additional postsynaptic AMPAR by exocytosis, a process called long-term potentiation (LTP). Low-frequency stimulation (LFS) on the other hand induces smaller, more prolonged Ca^2+^ increases which lead to synaptic weakening through postsynaptic AMPAR endocytosis which is termed long-term depression (LTD) [23]. Plasticity of glutamatergic synapses is essential for brain development [24] and activity-dependent cognitive processes like learning and memory [25]. Malfunctions in synaptic plasticity are thought to underlie ID [24]. Both LTP and LTD have been most intensively studied in the hippocampus, a brain region involved in spatial and declarative learning and memory [26], where both mechanisms were shown to be impaired in different models of ID [27–30]. So far, very little is known about the role of Slack in hippocampal synaptic plasticity.

Using electrophysiological, molecular, biochemical and functional imaging approaches, we find severely impaired synaptic function in the hippocampus of infant and milder deficits in adult *Slack*^*−/−*^ mice. Hence, we propose that early Slack channel dysfunction contributes to ID by causing abnormal synaptic development and synaptic plasticity.

## Materials and methods

### Animals

All experimental procedures were conducted in accordance with the animal protection laws in Germany and were approved by the local Ethics Committee for Animal Research (Regierungspräsidium Tübingen). Animals were maintained on a 12/12 h light/dark cycle (lights on 6 a.m.–6 p.m.) with access to food and water ad libitum. Slack-deficient mice (B6.129-Kcnt1^tm1Ruth^/RuLu) were generated by targeted ablation of *Kcnt1* in murine embryonic stem cells in combination with a common Cre/loxP-system as described previously [16] and backcrossed for at least nine generations to a C57BL/6N background. Pup genotypes were determined by PCR. Primer sequences are listed in Table S7. All experiments were performed with animals from both sexes. For experiments with adult animals, litter-matched offspring from heterozygous (*Slack*^+/−^) parental animals were used. Due to high numbers of pups required and the limited time available for genotyping, age-matched offspring from separate matings of homozygous *Slack*^+/+^ and *Slack*^−/−^ mice was used for dissociated cultures and experiments with P6 to P14 mice.

### Antibodies and reagents

All primary antibodies used in the present study are listed in Table S8. All secondary antibodies were isotype-specific and conjugated to Alexa Fluor 488- or Alexa Fluor 555 (Thermo Fisher Scientific). dl-2-Amino-5-phosphonopentanoic acid sodium salt (AP-5), (s)-3,5-Dihydroxyphenylglycine (DHPG), 2,3-Dioxo-6-nitro-1,2,3,4-tetrahydrobenzo[f]quinoxaline-7-sulfonamide (NBQX), N-Methyl-d-Aspartate (NMDA) and Ro 25-6981 were from Tocris, Picrotoxin (PiTX) and glutamic acid from Sigma-Aldrich. DNA oligonucleotide primers used in this study are listed in Table S7. If not specifically stated, all other reagents were of standard quality and from the usual vendors.

### Electrophysiology

Extracellular fEPSP recordings were performed according to standard methods as previously described [31, 32]. Mice were anaesthetized with CO_2_, decapitated and brains put into ice cold dissection buffer (in mM: 127 NaCl, 1.9 KCl, 26 NaHCO_3_, 1.2 KH_2_PO_4_, 10 d-glucose, 2 MgSO_4_, and 1.1 CaCl_2_, saturated with 5% CO_2_ and 95% O_2,_ final pH 7.4). The cerebellum was removed and 400 µm thick forebrain slices were cut with a vibratome (Leica VT 1000S) and subsequently maintained in artificial cerebrospinal fluid (ACSF, in mM: 127 NaCl, 1.9 KCl, 26 NaHCO_3_, 1.2 KH_2_PO_4_, 2.2 CaCl_2_, 1 MgSO_4_ and 10 d-glucose, oxygenated with 95% O_2_ plus 5% CO_2_, final pH 7.4) for 1 h at 30 °C and then for up to 5 h at room temperature. Slices were transferred into a submerged type recording chamber (Warner Instruments) constantly perfused with oxygenated ACSF at 30 °C. For LTD recordings, ACSF was supplemented with 50 µM PiTX. Stimulation (bipolar concentric, TM53CCINS, WPI) and recording (ACSF-filled glass pipettes, 2–3 MΩ) electrodes were positioned in the *stratum radiatum* to record Schaffer collateral fEPSPs. Signals were amplified with an Axopatch 200B amplifier (Molecular Devices), digitized at 5 kHz with a LIH 8 + 8 (HEKA) and recorded using WinWCP from the Strathclyde Electrophysiology Suite. Stimuli (100 µs) were delivered through a stimulus isolator (WPI).

NMDAR-dependent LTD and LTP were elicited using low-frequency stimulation (LFS) of 900 stimuli at 1 Hz (15 min) and high-frequency stimulation (HFS) of 100 stimuli at 100 Hz (1 s), respectively. mGluR-dependent LTD was accomplished by wash-in of 100 µM DHPG for 10 min. The same stimulus intensity was used during baseline recording (0.067 Hz), LFS, HFS and wash-in of DHPG. The baseline was determined by the average of fEPSP initial slopes from the period before LFS, HFS, or wash-in of DHPG. The respective level of LTD or LTP was determined by the average of fEPSP initial slopes from the period between 45 and 60 min after the LFS, HFS or wash-in of DHPG. Before eliciting LTD, LTP or wash-in of DHPG, each slice was used to record input–output relation (IOR) for stimulus intensities of 25–150 µA as well as paired-pulse facilitation (PPF) for inter-stimulus intervals of 10 ms, 20 ms, 50 ms, 100 ms, 200 ms, and 500 ms (at the same stimulation strength as LTD and LTP recordings).

NMDAR-mediated fEPSP were isolated by simultaneously removing extracellular Mg^2+^ and inhibiting AMPAR-mediated transmission with NBQX (10 μM). The NMDAR blocker AP-5 (100 µM) was routinely added at the end of each experiment to assure that recorded fEPSP were indeed due to NMDAR only.

Four traces were averaged for each data point. Data were analyzed and processed using Clampfit 10 (Molecular Devices) and Microsoft Excel. fEPSP decay time constant τ_decay_ of signals elicited by 150 µA was determined by fitting a single exponential decay function between the negative peak and the end of the trace using the built-in function of Clampfit. Statistics and visualization were performed with GraphPad Prism. Results between conditions were statistically compared using one-way ANOVA and Sidak's multiple comparison Test to compare baseline vs. LTD or LTP for both genotypes as well as LTD or LTP between genotypes.

### Golgi staining

Golgi staining was performed using the *super*Golgi Kit (Bioenno Tech) according to the manufacturer's instructions. Forebrains were removed and immersed in potassium dichromate solution for 8 days, before incubation in post-incubation buffer for 2 days. 150 µm vibratome sections were subsequently mounted on glass slides, washed in PBS with 0.01 M Triton X-100 (PBST) and stained with ammonium hydroxide before immersion in post-staining buffer. Slices were washed with PBST again, dehydrated with ethanol and xylene and coverslipped with Depex. Transmitted light Z-stacks were imaged on a Zeiss AxioScope and analyzed by a sample blinded operator using ImageJ [33]. Concentric circles for Sholl analysis were drawn by the ImageJ extension ConcentricCircles, crossings were counted manually. Spines were counted manually using ImageJ’s Cell Counter plugin.

### Quantitative RT-PCR

Real-time quantitative PCR (RT-qPCR) experiments were performed as previously described [34]. Hippocampi were rapidly dissected and homogenized by hand disperser in PeqGOLD RNApure (Peqlab). Total RNA was extracted under RNase-free conditions according to the manufacturer’s instructions. To minimize genomic DNA, extracted RNA samples were DNAse treated for 30 min and quantified with a NanoPhotometer (Implen). 500 ng RNA was used for first strand cDNA synthesis using iScript cDNA Synthesis Kit (Bio-Rad Laboratories). Real-time RT–PCR was performed on a CFX Connect Real-Time PCR Detection System (Bio-Rad) with SsoAdvanced Universal SYBR Green Supermix (Bio-Rad). PCR reactions were performed in triplicate by incubating at 95 °C for 2 min, followed by 40 cycles of 5 s at 95 °C and 20 s at 58 °C. Water controls and -RT samples (where reverse transcriptase was omitted during the first strand synthesis) were included to ensure specificity of the primer pairs. Relative expression of target gene levels was determined using the comparative 2^−ΔΔCt^ method. Expression levels where normalized to hypoxanthine–guanine phosphoribosyltransferase (HPRT). Primers specific to target RNAs were selected using Primer3 software. Sequences for all primers used in this study are listed in Table S7.

### Biochemical fractionation and immunoblotting

Subcellular fractionation was performed as described earlier [35]. Seven whole forebrains were pooled from P9 and 4–8 hippocampi from adult *Slack*^+*/*+^ or *Slack*^*−/−*^ mice. Tissue was removed and shock frozen in liquid N_2_ before Potter–Elvehjem homogenization in 0.32 M sucrose, 1 mM Tris pH 7.4, 1 mM MgCl_2_ with protease inhibitors (in µg/ml; 1 phenylmethanesulfonyl fluoride, 1 pepstatin A, 10 leupeptin, 20 aprotinin). Lysate was spun 15 min at 1400×*g*. Supernatant was collected and saved. The pellet was homogenized with more sucrose solution and large insoluble debris and the nuclear fraction were removed by centrifugation at 710×*g*. The supernatants were pooled and an aliquot saved as the postnuclear supernatant (S1) fraction. The remaining S1 was centrifuged 13,800×*g*, and the pellet collected as the membrane-enriched fraction (P2). The P2 fraction was resuspended in 0.32 M sucrose solution without MgCl_2_ and layered over 0.85/1.0/1.25 M sucrose gradient, spun at 82,500×*g* for 2 h. Synaptosomal enriched fraction (syn) was collected between 1.0/1.25 M sucrose layers. Triton X-100 was added to syn to a final concentration of 1% and incubated for 15 min at 4 °C. The pellet was collected after 35,000×*g* spin for 30 min. The pellet was resuspended in 0.32 M sucrose, 1 mM Tris pH 7.4 with protease inhibitors, placed over another sucrose gradient (1.0/1.5/2.0 M), centrifuged at 22,500×*g* for 2 h, and second gradient layer collected between 1.5/2.0 M sucrose interface. This fraction was exposed to 1% Triton X-100 again and a final PSD pellet collected after centrifugation at 100,000×*g* for 1 h, which was resuspended via sonication to obtain the PSD enriched fraction (PSD).

Protein concentration in the obtained fractions was measured using a Bradford assay. 8–12 μg total protein were incubated with SDS sample buffer at 95 °C for 5 min, separated by electrophoresis in 10% polyacrylamide gels, transferred to polyvinylidene fluoride (PVDF) membranes, probed with the indicated primary antibodies (Table S8) and detected with fluorescently labelled secondary antibodies. Immunosignals were visualized using an Amersham Imager 600 (General Electrics) and densitometrically analyzed by ImageJ.

### Dissociated neuronal cultures and Ca^2+^ imaging

Primary hippocampal neurons were cultured from P0 *Slack*^+*/*+^ and *Slack*^*−/−*^ as previously described [36]. Briefly, hippocampi were dissected and freed from meninges in dissection medium (DM) consisting of Hank’s Balanced Salt Solution (HBSS, Invitrogen #14175095) supplemented with 1 mM sodium pyruvate, 0.1% (wt/vol) Glucose and 10 mM HEPES. After washing thrice with DM, tissue was trypsinized 0.25% (wt/vol) in DM for 20 min at 37 °C. Subsequently, 0.1% (wt/vol) Deoxyribonuclease I (Sigma #DN25) were added for 5 min at room temperature, before tissue was washed twice with DM and trypsin was inactivated with plating media (PM; BME medium, Invitrogen # 21010046) supplemented with 10% FCS, 0.45% (wt/vol) glucose, 1 mM sodium pyruvate, 2 mM glutamine and 100 U/ml penicillin/streptomycin. Cells were dissociated by trituration with a fire-polished Pasteur pipette, counted and seeded on coverslips precoated with 0.5% (wt/vol) poly-l-lysine (Sigma #P2636) in PM. After 2 h, medium was changed to serum-free maintenance medium (Neurobasal; Invitrogen # 21103049) supplemented with B-27 (Invitrogen; # 17504044), 2 mM glutamine and 100 U/ml penicillin/streptomycin. Cells were maintained at 37 °C in a humidified environment with 5% CO_2_/95% air. Half of the culturing medium was replaced every 3–4 days.

After 8 days in vitro (8 DIV), intracellular calcium concentration ([Ca^2+^]_i_) changes in response to 60 s glutamate stimulation were determined by Ca^2+^ measurement with the ratiometric dye fura-2AM. To this end, cells were loaded with 2.5 µM fura-2AM (Merck KGaA, Darmstadt, Germany) in 1 ml fACSF (in mM: 138 NaCl, 5 KCl, 2 CaCl_2_, 10 glucose, 10 HEPES, final pH 7.4) for 45 min. Dishes were then placed in a perfusion chamber mounted on a Zeiss Axiovert S100 inverted microscope and cultures were perfused with prewarmed (37 °C) fACSF solution to remove the extracellular dye. Solution exchange and cell stimulation during data acquisition were performed using the PC30 perfusion chamber (NGFI GmbH, Graz, Austria) connected to a gravity-based perfusion system (NGFI GmbH). After recording of baseline [Ca^2+^]_i_ levels, changes in [Ca^2+^]_i_ concentration of individual neurons were monitored during 60 s glutamate stimulation in presence of the indicated pharmacological modulators. Illumination at different wavelengths was achieved by a shutter and fluorescent images were acquired with a Spot Inside camera (Visitron, Puchheim, Germany). Relative [Ca^2+^]_i_ levels were calculated from the ratio of background-corrected fura-2AM emission (520 nm) at the two different excitation wavelengths (340 and 380 nm). VisiView software (Visitron, Puchheim, Germany) was used for image acquisition. Data was analyzed using Microsoft Excel. Graphpad Prism was used for statistical analysis and visualization.

### Chemical LTD (cLTD)

Mice were anaesthetized with CO_2_, decapitated and brains put into ice cold sucrose dissection buffer (SACSF, in mM: 254 sucrose, 1.9 KCl, 1.2 KH_2_PO_4_, 26 NaHCO_3_, 10 d-glucose, 2 MgSO_4_ and 1.1 CaCl_2_, saturated with 5% CO_2_ and 95% O_2,_ final pH 7.4). Hippocampi were extracted. 400 μm transversal slices were prepared using a McIlwain Tissue Chopper and incubated in oxygenated ACSF (see above) for 30 min. cLTD was induced by adding 100 µM freshly prepared NMDA for 5 min. Slices were subsequently stored in NMDA-free ACSF for 10 min before snap freezing in liquid N_2_. Frozen slices were quickly lysed with 100 μl ice-cold RIPA buffer (in mM: 50 Tris, 150 NaCl, 5 EGTA, 10 EDTA, final pH 7.4) containing protease inhibitors (see above) as well as 1% NP-40, 10% glycerol, 0.05% sodium dodecyl sulfate (SDS), 0.4% deoxycholate including phosphatase inhibitors (Sigma Phosphatase Inhibitor Cocktail 2 & 3) using a hand disperser (Polytron). Homogenates were cleared by centrifugation and protein concentration was measured using a Bradford assay. 30 μg sample were separated by SDS-PAGE and detected by Western blot using the indicated antibodies (Table S8) as described above. After detection of phospho-specific signals, membranes were stripped using Re-blot Plus Strong Antibody Stripping Solution (Millipore) before incubation with GluA1-specific antibodies and detection with secondary antibodies labelled with a fluorescent dye different from the one used for detection of phospho signals (all antibodies listed in Table S8).

### Mass spectrometry

Biochemical fractionation was performed as described above. Fractionated samples were precipitated with chloroform and methanol followed by trypsin digestion as described before [37]. LC–MS/MS analysis was performed on Ultimate3000 nanoRSLC systems (Thermo Scientific) coupled to an Orbitrap Fusion Tribrid mass spectrometer (Thermo Scientific) by a nano spray ion source. Tryptic peptide mixtures were injected automatically and loaded at a flow rate of 30 μl/min in 0.1% trifluoroacetic acid in HPLC-grade water onto a nano trap column (300 μm i.d. × 5 mm pre-column, packed with Acclaim PepMap100 C18, 5 μm, 100 Å; Thermo Scientific). After 3 min, peptides were eluted and separated on the analytical column (75 μm i.d. × 25 cm, Acclaim PepMap RSLC C18, 2 μm, 100 Å; Thermo Scientific) by a linear gradient from 2 to 30% of buffer B (80% acetonitrile and 0.08% formic acid in HPLC-grade water) in buffer A (2% acetonitrile and 0.1% formic acid in HPLC-grade water) at a flow rate of 300 nl/min over 117 min. Remaining peptides were eluted by a short gradient from 30 to 95% buffer B in 5 min. Analysis of the eluted peptides was done on an LTQ Fusion mass spectrometer. From the high-resolution MS pre-scan with a mass range of 335 to 1500, the most intense peptide ions were selected for fragment analysis in the ion trap using a high-speed method if they were at least doubly charged. The normalized collision energy for HCD was set to a value of 27 and the resulting fragments were detected with normal resolution. The lock mass option was activated; the background signal with a mass of 445.12003 was used as lock mass [38]. Every ion selected for fragmentation was excluded for 20 s by dynamic exclusion. MS/MS data were analyzed using the MaxQuant software (version 1.6.1.0) [39, 40]. As a digesting enzyme, Trypsin/P was selected with maximal 2 missed cleavages. Cysteine carbamidomethylation was set for fixed modifications, and oxidation of methionine and N-terminal acetylation were specified as variable modifications. The data were analyzed by label-free quantification with the minimum ratio count of 3. The first search peptide tolerance was set to 20, the main search peptide tolerance to 4.5 ppm and the re-quantify option was selected. For peptide and protein identification the mouse subset of the SwissProt database (release 2014_04) was used and contaminants were detected using the MaxQuant contaminant search. A minimum peptide number of 2 and a minimum length of 7 amino acids was tolerated. Unique and razor peptides were used for quantification. The match between run option was enabled with a match time window of 0.7 min and an alignment time window of 20 min. The statistical analysis including ratio, t test and significance B calculation was done using the Perseus software (version 1.5.5.3, [41].

### Experimental design and statistical analysis

Data were analyzed using Graphpad Prism version 8. All statistical information and sample numbers can be found in the results section, Tables S1–S6 and figure legends to Figures S1–S4. In figures, significance is indicated by asterisks (*p < 0.05, **p < 0.01, ***p < 0.001). n.s. denotes non-significant results (*p* > 0.05). All data are presented as mean ± SEM.

## Results

### Infant ***Slack***^***−/−***^ lack NMDAR-dependent hippocampal plasticity

**Fig. 1:**
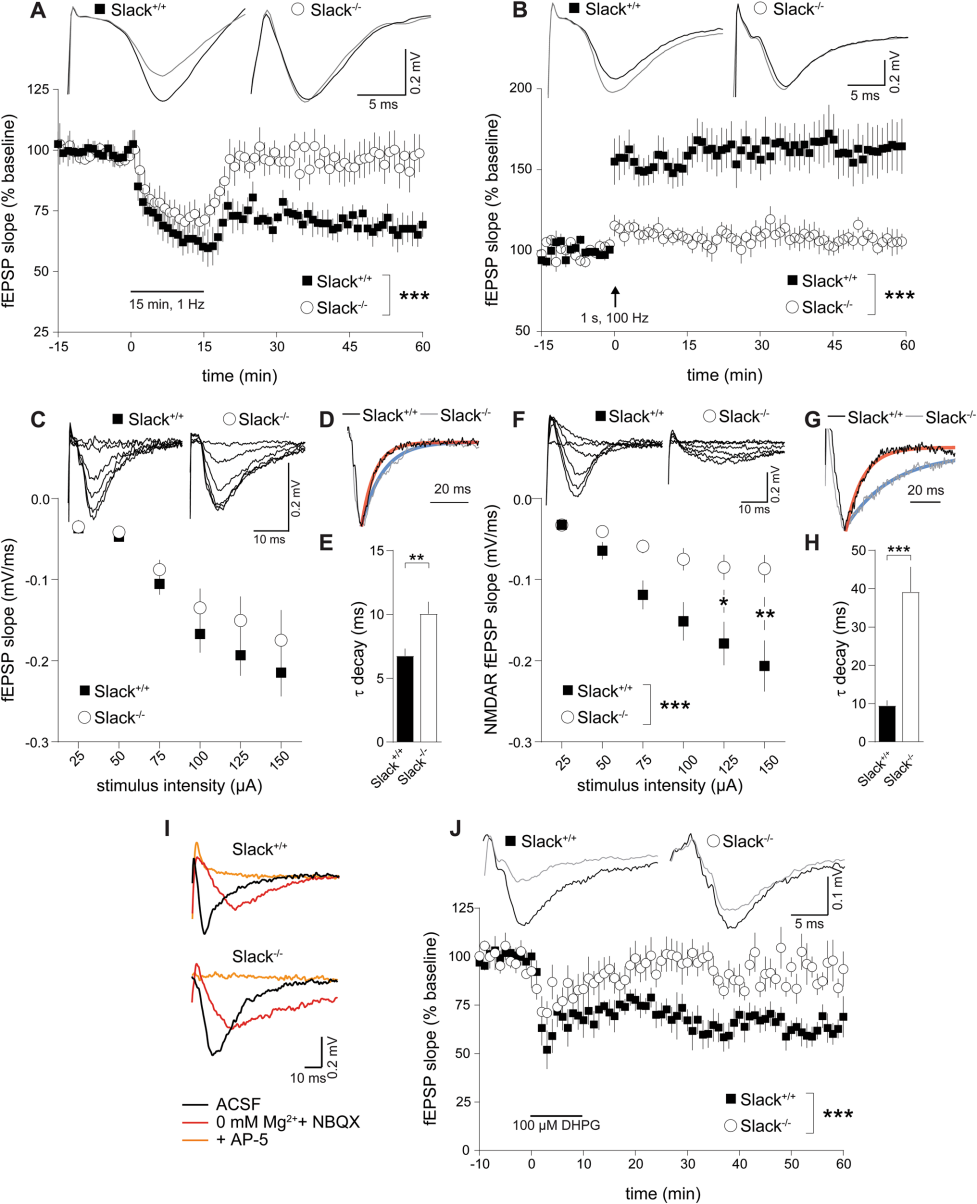
Lack of synaptic plasticity and reduced NMDAR-mediated transmission in infant *Slack*^*−/−*^*.* Schaffer-collateral fEPSP initial slopes recorded from acute forebrain slices of P6 to P14 *Slack*^+*/*+^ and *Slack*^*−/−*^. **A**, **B** 15 min/1 Hz low-frequency stimulation (LFS) led to significant LTD in *Slack*^+*/*+^ (n slices/animals = 10/5) but not *Slack*^*−/−*^ (*n* = 10/4) (**A**), while 100 Hz/1 s high-frequency stimulation (HFS) induced significant LTP in *Slack*^+*/*+^ (*n* = 13/8) but not *Slack*^*−/−*^ (*n* = 9/4) (**B**). Top: Representative traces before (black) and after (gray) LTD induction. **C** Averaged initial fEPSP slopes recorded at stimulation intensities of 25–150 µA in 25 µA increments were not different between *Slack*^+*/*+^ (*n* = 12/4) and *Slack*^*−/−*^ (*n* = 7/3). Top: Representative traces. **D**, **E** Single exponential decay fit of responses to 150 µA stimulation revealed significantly slower decay time constants of fEPSP in *Slack*^*−/−*^ (*n* = 12) compared to *Slack*^+*/*+^ (*n* = 8). **D** Representative fEPSP in response to 150 µA stimulation, normalized to peak. Shape of single exponential decay fit function is indicated in red for *Slack*^+*/*+^ and in blue for *Slack*^*−/−*^*.* **E** τ_decay_ from traces elicited by 150 µA stimulation. **F** Initial slopes of NMDAR-mediated fEPSP, isolated in Mg^2+^-free ACSF containing the AMPAR antagonist NBQX (10 µM), recorded at stimulation intensities of 25–150 µA in 25 µA increments were significantly lower in *Slack*^*−/−*^ (*n* = 9/2) compared to *Slack*^+*/*+^ (*n* = 18/ 3). Top: Representative traces. **G**–**I** Single exponential decay fit of responses to 150 µA stimulation revealed significantly slower decay time constants of NMDAR fEPSP in *Slack*^*−/−*^ (*n* = 9) compared to *Slack*^+*/*+^ (*n* = 18). **G** Representative NMDAR fEPSP in response to 150 µA stimulation (in Mg^2+^-free ACSF plus NBQX), normalized to peak. Shape of single exponential decay fit function is indicated in red for *Slack*^+*/*+^ and in blue for *Slack*^*−/−*^*.* **H** τ_decay_ from NMDAR fEPSP traces elicited by 150 µA stimulation. **I** Representative fEPSP in normal ACSF (black, corresponds to traces in **C**), Mg^2+^-free ACSF with NBQX (red, corresponds to traces in **F**) and after addition of the NMDAR antagonist AP-5 (100 µM, orange). **J** Superfusion of 100 µM DHPG for 10 min induced significant mGluR-dependent LTD in *Slack*^+*/*+^ (*n* = 8/2), but not *Slack*^*−/−*^ (*n* = 10/3). Top: Representative traces recorded before (black) and after (gray) LTD induction by DHPG. Statistics: Two-way ANOVA with Sidak's multiple comparison test (**A**, **B**, **C**, **F**), Student's t test (**E**). All bar diagrams presented as means ± SEM. See also Table S1

We recently reported impaired cognitive flexibility of adult *Slack*^*−/−*^ in learning tasks [5] associated with LTD in hippocampal Schaffer-collateral CA1 to CA3 synapses [42, 43]. Therefore, we characterized synaptic plasticity in *Slack*^*−/−*^ by measuring excitatory postsynaptic potentials (EPSP) as extracellular field potentials (fEPSP) in hippocampal slices from postnatal day 6 (P6) to P14 mice. Infants were chosen because LTD can be masked in older animals [44, 45]. Additionally, this should allow discrimination between developmental and acute contributions of Slack on LTD induction, maintenance, and expression. Although GABAergic transmission in the mouse hippocampus is inhibitory by P7 [46], 50 µM of the GABA_A_ receptor antagonist picrotoxin (PiTX) were included to rule out altered excitatory or inhibitory influence of GABA on LTD experiments in the investigated age range. As expected, we could induce significant LTD in hippocampi derived from infant *Slack*^+*/*+^ but not *Slack*^*−/−*^ mice (Fig. 1A), which is in accordance with impaired reversal learning of adult *Slack*^*−/−*^ in the previously performed Morris Water Maze (MWM) [5]. As MWM acquisition performance, which is usually correlated to hippocampal LTP [47] was not affected in adult *Slack*^*−/−*^ [5], we choose a mild 1 s, 100 Hz HFS paradigm to elicit LTP. We could observe significant LTP in infant *Slack*^+*/*+^, but surprisingly not in *Slack*^*−/−*^ (Fig. 1B), suggesting that synaptic plasticity is more severely affected in infant than in adult *Slack*^*−/−*^.

### fEPSP decay is delayed in infant *Slack*^*−/−*^

Alterations of synaptic function in general [48] and in ID [49] are often accompanied by changes in dendritic branching or spine density. We, therefore, compared Golgi-stained CA1 pyramidal neurons from P9 *Slack*^+*/*+^ and *Slack*^*−/−*^. Basal dendrite branching was not different (Figure S1A) while apical dendrite branching was slightly, but significantly increased in *Slack*^*−/−*^ (Figure S1B). Synaptic spine density was similar in both genotypes (Figure S1C). Presynaptic facilitation of fEPSP (Figure S1D) and signal strength in relation to stimulus intensity (Fig. 1C), however, were not different between both genotypes, indicating unaltered transmission at Schaffer-collateral synapses between infant *Slack*^*−/−*^ and *Slack*^+*/*+^, possibly due to slightly increased apical branching in the absence of Slack. Interestingly, fEPSP in *Slack*^*−/−*^ decayed significantly slower than in *Slack*^+*/*+^ (Fig. 1D, E). This delayed repolarization might be due to lack of the hyperpolarizing Slack conductance in *Slack*^*−/−*^. Indeed, potassium channel inhibition was previously demonstrated to delay EPSP decay [50, 51].

### Infant *Slack*^*−/−*^ show reduced NMDAR-mediated transmission and lack mGluR LTD

We next assessed the extent of NMDAR-mediated synaptic transmission essential for LTD and LTP induction [21]. NMDAR-mediated fEPSP strength, isolated in the presence of the AMPA antagonist NBQX, was significantly reduced in P6-14 *Slack*^*−/−*^ (Fig. 1F) and decayed significantly slower than in *Slack*^+*/*+^ (Fig. 1G, H). Slowing of decay was even more pronounced for NMDAR-mediated fEPSP as for AMPAR-mediated total fEPSP (compare Fig. 1E–H). Any potential residual contribution of unblocked AMPAR to the recorded NMDAR fEPSP was excluded by adding the NMDAR antagonist AP-5 at the end of each recording to completely block the response (Fig. 1I). These results suggest that NMDAR signaling deficits lead to impaired LTD induction in *Slack*^*−/−*^. To investigate if LTD expression is affected as well, we used the selective group I metabotropic glutamate receptor (mGluR) agonist (*s*)-3,5-Dihydroxyphenylglycine (DHPG) to trigger hippocampal LTD independently of NMDAR [43]. DHPG induced significant Schaffer-collateral LTD in *Slack*^+*/*+^ but not *Slack*^*−/−*^ (Fig. 1J). Apparently, lack of LTD in infant *Slack*^*−/−*^ is not only caused by defective NMDAR-mediated LTD induction, but also involves either altered mGluR expression [52], or alternatively, impaired LTD expression mechanisms like AMPAR endocytosis and vesicular transport [53].

### GluN2B expression is reduced in infant *Slack*^*−/−*^

**Fig. 2:**
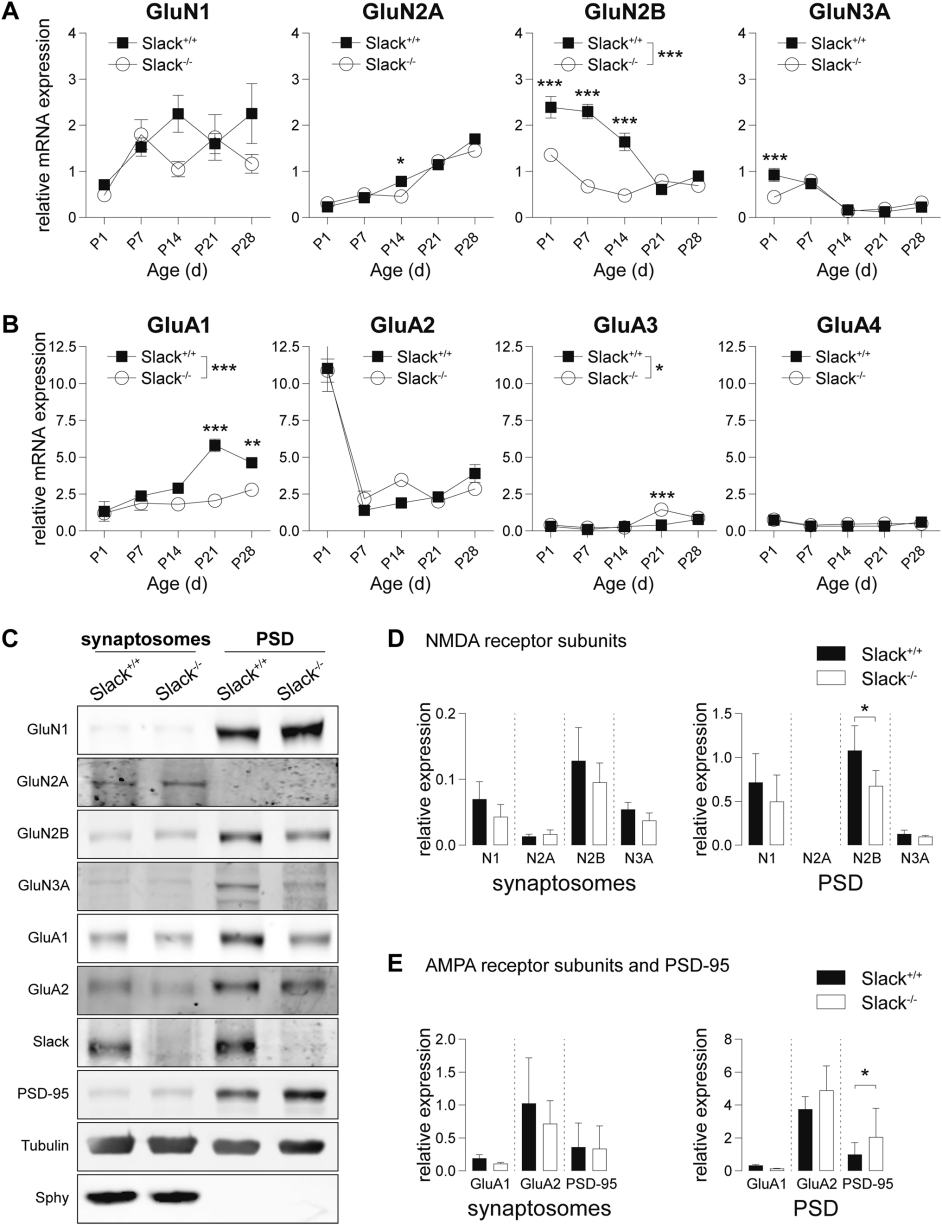
Reduced GluN2B expression in infant *Slack*^*−/−*^ hippocampi. **A**, **B** mRNA from *Slack*^+*/*+^ and *Slack*^*−/−*^ hippocampi isolated weekly from P1 to P28 for quantitative RT-PCR analysis. Values were normalized to HPRT. For each value, at least *n* = 3 independent RNA isolations were performed of animals from different litters. **A** GluN1 transcripts increased between P1 and P7, and remained constant thereafter in both genotypes. GluN2A transcripts increased in parallel between P1-P28 and were significantly lower in *Slack*^*−/−*^ only at P14. High postnatal GluN2B in *Slack*^+*/*+^ decreased between P1-P21 while the same trend was seen in *Slack*^*−/−*^ albeit at significantly lower levels. At P1 GluN3A transcripts were reduced in *Slack*^*−/−*^, but equally decreased in both genotypes between P7 and P14. **B** GluA1 transcripts were significantly lower in *Slack*^*−/−*^ than *Slack*^+*/*+^ at P21 and P28. GluA2 steeply decreased between P1 and P7 and stayed level afterwards in both genotypes. GluA3 transcripts were generally very low, but significantly reduced in *Slack*^*−/−*^ at P21. GluA4 was low and not different between *Slack*^+*/*+^ and *Slack*^*−/−*^. **C** Representative immunoblots of biochemical fractions derived from P9 *Slack*^+*/*+^ and *Slack*^*−/−*^ forebrains. Separated synaptosomal and PSD enriched fractions were probed with antibodies for GluN1, GluN2A, GluN2B, GluN3A, GluA1, GluA2 and Slack. Controlling for successful fractionation, PSD-95 was enriched in PSD and de-enriched in synaptosomes. Pre-synaptic synaptophysin (Sphy), is absent from PSD but enriched in synaptosomes. **D**, **E** Quantification of NMDA (**D**) as well as AMPA receptor subunits and PSD-95 (**E**) protein band intensities normalized to α-tubulin in synaptosomal (left) and PSD (right) enriched fractions. GluN2B is significantly reduced in *Slack*^*−/− *^PSD enriched fractions. Each value corresponds to at least *n* = 3 independent preparations of nine pooled forebrains. GluN2A was not detectable in PSD. Statistics: Two-way ANOVA with Sidak's multiple comparison test (**A**, **B**), Student's t test (**D**, **E**). All bar diagrams presented as means ± SEM. See also Table S2

Function of heterotetrameric NMDAR [54] and AMPAR [23] is crucially determined by their respective subunit composition. Hippocampal NMDAR predominantly contain two mandatory GluN1 subunits and two GluN2 subunits one of which can be replaced by GluN3A in very young animals [54]. Hippocampal AMPAR are mainly comprised of GluA1 and GluA2 subunits with minor amounts of both GluA3 and GluA4 [23]. Altered NMDAR and AMPAR subunit stoichiometry might thus explain differences in synaptic plasticity between *Slack*^+*/*+^ and *Slack*^*−/−*^. Therefore, we examined glutamate receptor subunit composition during the first postnatal month using quantitative RT-PCR (Fig. 2A, B). NMDAR subunit transcription in *Slack*^+*/*+^ corresponded to previous observations [54] including the developmental switch from GluN2B to GluN2A between P1 to P28 (Fig. 2A). In *Slack*^*−/−*^, however, GluN2B transcripts levels were significantly lower than in *Slack*^+*/*+^ directly after birth and remained low up to P28 (Fig. 2A), including the P6-14 time range examined in electrophysiological recordings (Fig. 1). In the P1-14 time-window, transcript levels of all AMPAR subunits assessed were similar between genotypes (Fig. 2B). After P14, however, we find a Slack-dependent induction of GluA1 transcript levels (Fig. 2B). The physiological consequences of this increase remain unknown, as synaptic transmission was only monitored in P6-14 animals (Figs. 1 and S1). In contrast to NMDAR and AMPAR subunits, we could not find developmental regulation of Slack transcription in *Slack*^+*/*+^ brains (Figure S2A), nor was there compensatory transcriptional regulation of the closely related Na^+^-activated K^+^ channel Slick in *Slack*^*−/−*^ (Figure S2B).

Next, we isolated total synaptosomes and postsynaptic density (PSD) enriched protein fractions from forebrains of juvenile mice to investigate whether glutamate receptor protein levels correspond to the observed changes in transcript expression (Fig. 2C). Indeed, GluN2B abundance in PSD, but not in synaptosomal enriched fractions from *Slack*^*−/−*^ was lower (Fig. 2D). Additionally, we identified elevated PSD-95 levels in PSD enriched fractions from *Slack*^*−/−*^ (Fig. 2E) indicative of compensation for reduced Slack and GluN2B levels (Fig. 2A), but also a potential consequence of slightly increased dendritic branching (Figure S1B). Interestingly, Slack presence could be verified in all biochemical fractions isolated during synaptosome and PSD purification, illustrating the channel's wide expression across different neuronal compartments (Figure S2C). Importantly, lack of postsynaptic GluN2B possibly explains impaired NMDAR-dependent synaptic plasticity in *Slack*^*−/−*^ (Fig. 1) as hippocampal GluN2B deletion was shown to hamper LTP and particularly LTD ([55, 56], but also see [57]). The slowing of NMDAR-mediated fEPSP inactivation kinetics in *Slack*^*−/−*^ cannot be explained by altered NMDAR subunit composition, as heterologously expressed GluN2A-containing NMDAR usually inactivate faster than GluN2B-containing receptors [54]. As decay of AMPAR-mediated fEPSP in *Slack*^*−/−*^ is also slower than in *Slack*^+*/*+^ (Fig. 1E), it seems most likely, that the lack of the hyperpolarizing Slack conductance in *Slack*^*−/−*^ postsynaptic neurons leads to delayed repolarization of the membrane, but the underlying mechanisms are yet unknown.

### Ca^2+^ entry in *Slack*^*−/−*^ neurons is less sensitive to the GluN2B-specific inhibitor Ro 25–6981

**Fig. 3:**
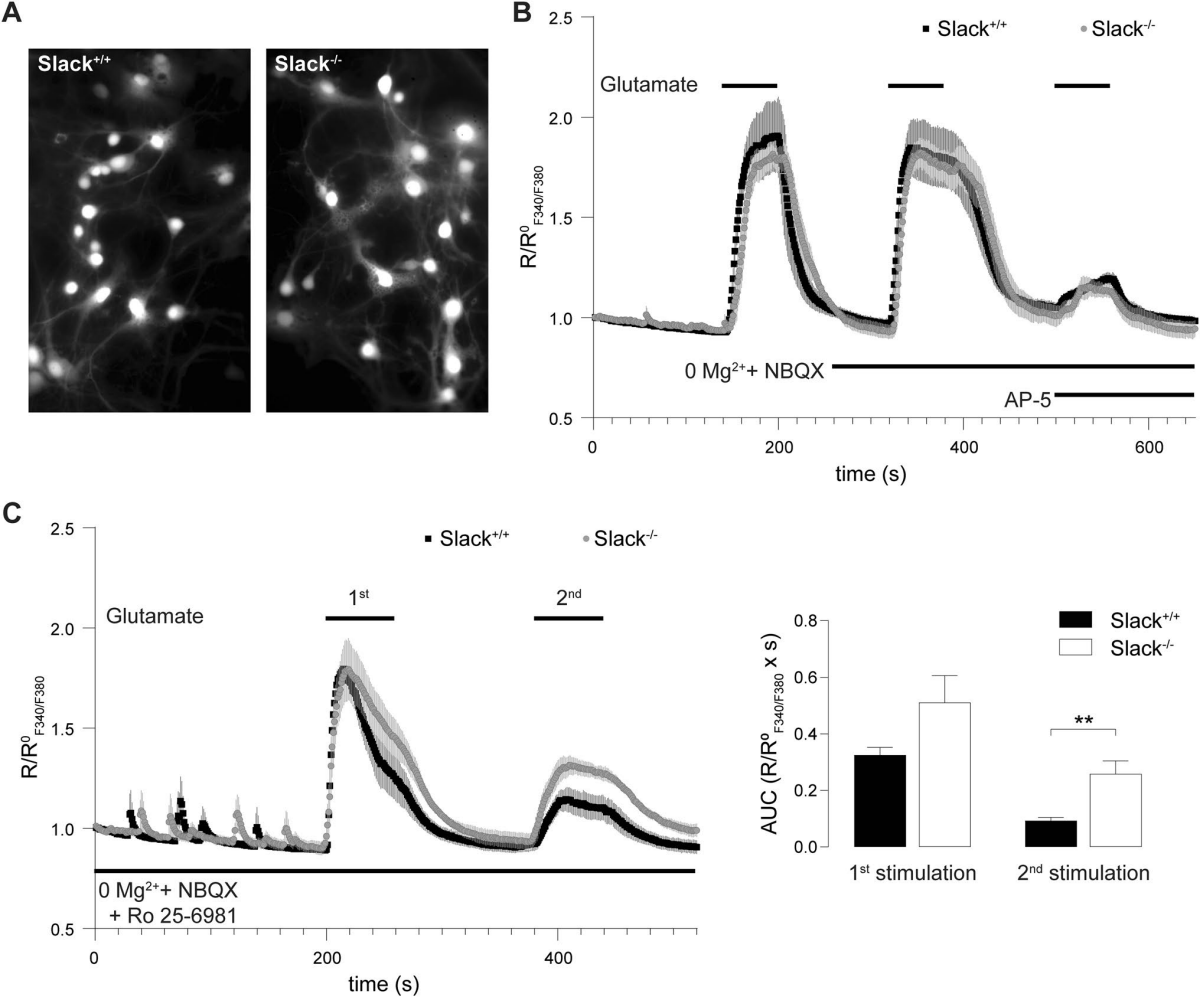
Reduced GluN2B-mediated Ca^2+^ entry in dissociated hippocampal *Slack*^*−/−*^ neurons. **A** 8 DIV dissociated hippocampal neuronal cultures prepared from newborn pups were loaded with the Ca^2+^-sensitive dye fura-2AM. **B** Averaged time course of ratio between fluorescence intensities at 340 nm and 380 nm (R_F340/F380_) in *Slack*^+*/*+^ (*n* = 39 cells) and *Slack*^*−/−*^ (*n* = 40 cells) neurons treated with 10 µM glutamate for 60 s. Ca^2+^-entry through NMDAR was isolated in Mg^2+^-free ACSF containing 10 µM NBQX. Application if 100 µM AP-5 quantitatively abolished the remaining glutamate-stimulated Ca^2+^-entry. **C** Left panel: Averaged time course of R_F340/F380_ in *Slack*^+*/*+^ (*n* recordings/cultures/cells = 6/2/90) and *Slack*^*−/−*^ (*n* = 6/3/90) neurons in Mg^2+^-free ACSF containing 10 µM NBQX in presence of the specific blocker of GluN2B-containing NMDAR Ro 25–6981 (1 µM). Cultures were stimulated twice with 10 µM glutamate for 60 s to allow activity-dependent inhibition of GluN2B-containing NMDAR. Right panel: Area under the curve (AUC) of changes in R_F340/F380_ elicited by the 1st glutamate stimulation was not different between *Slack*^+*/*+^ and *Slack*^*−/−*^ neurons, but significantly reduced in *Slack*^+*/*+^ compared to *Slack*^*−/−*^ in response to the 2nd glutamate stimulation, which, compared to the 1st glutamate exposure resulted in lower NMDAR-mediated Ca^2+^ signals. Statistics: Student's t test (**C**). All diagrams presented as means ± SEM. See also Table S3

To test if reduced GluN2B expression in *Slack*^*−/−*^ leads to functional changes in neurons, we recorded NMDAR-mediated intracellular calcium concentration ([Ca^2+^]_i_) changes in dissociated hippocampal neuronal cultures from *Slack*^+*/*+^ and *Slack*^*−/−*^ as surrogate (Fig. 3A). Application of 10 µM glutamate for 60 s led to comparable [Ca^2+^]_i_ increases in *Slack*^+*/*+^ and *Slack*^*−/−*^. When NMDAR-mediated changes in [Ca^2+^]_i_ were isolated by omitting Mg^2+^ and adding the AMPAR antagonist NBQX, peak [Ca^2+^]_i_ levels were similar, but decayed slower than in regular recording solution in both genotypes. As expected, additional application of the NMDAR antagonist AP-5 quantitatively abolished glutamate-induced increases in [Ca^2+^]_i_. The remaining signal does not differ between *Slack*^*+/+*^ and *Slack*^*−/−*^ and most likely represents mGluR-invoked Ca^2+^ entry (Fig. 3B). Although there are fundamental methodical differences between experiments in Fig. 1F (acute slices and electrophysiology) and Fig. 3B (dissociated neuronal cultures and fluorescent dye calcium imaging), these results suggest that reduced amplitudes of NMDAR-mediated fEPSP in *Slack*^*−/−*^ (Fig. 1F) cannot be explained by Ca^2+^-influx through NMDAR in response to glutamate stimulation in individual cells (Fig. 3B). So far, we can only speculate that changes in some other, as of yet unknown, conductance are the underlying reason for this observation. Glutamate stimulation in the absence of Mg^2+^ and in the presence of NBQX and the GluN2B-specific NMDAR antagonist Ro 25–6981 [58] led to an increase in [Ca^2+^]_i_, that was initially comparable between *Slack*^+*/*+^ and *Slack*^*−/−*^, but seemed to decay faster in *Slack*^+*/*+^ (Fig. 3C). As NMDAR antagonism of Ro 25–6981 is activity-dependent [58], a second glutamate stimulation was carried out. Consistent with lower GluN2B expression in *Slack*^*−/−*^ (Fig. 2), this 2nd exposure to glutamate provoked stronger [Ca^2+^]_i_ changes in *Slack*^*−/−*^ than *Slack*^+*/*+^ (Fig. 3C) corresponding to reduced block by Ro 25-6981 in *Slack*^*−/−*^. This indicates that GluN2B contributes to NMDAR-mediated Ca^2+^ influx in *Slack*^*−/−*^ to a lower degree than in *Slack*^+*/*+^.

### *Slack*^*−/−*^ deficiency impairs dephosphorylation of GluA1 S845 after LTD induction

**Fig. 4:**
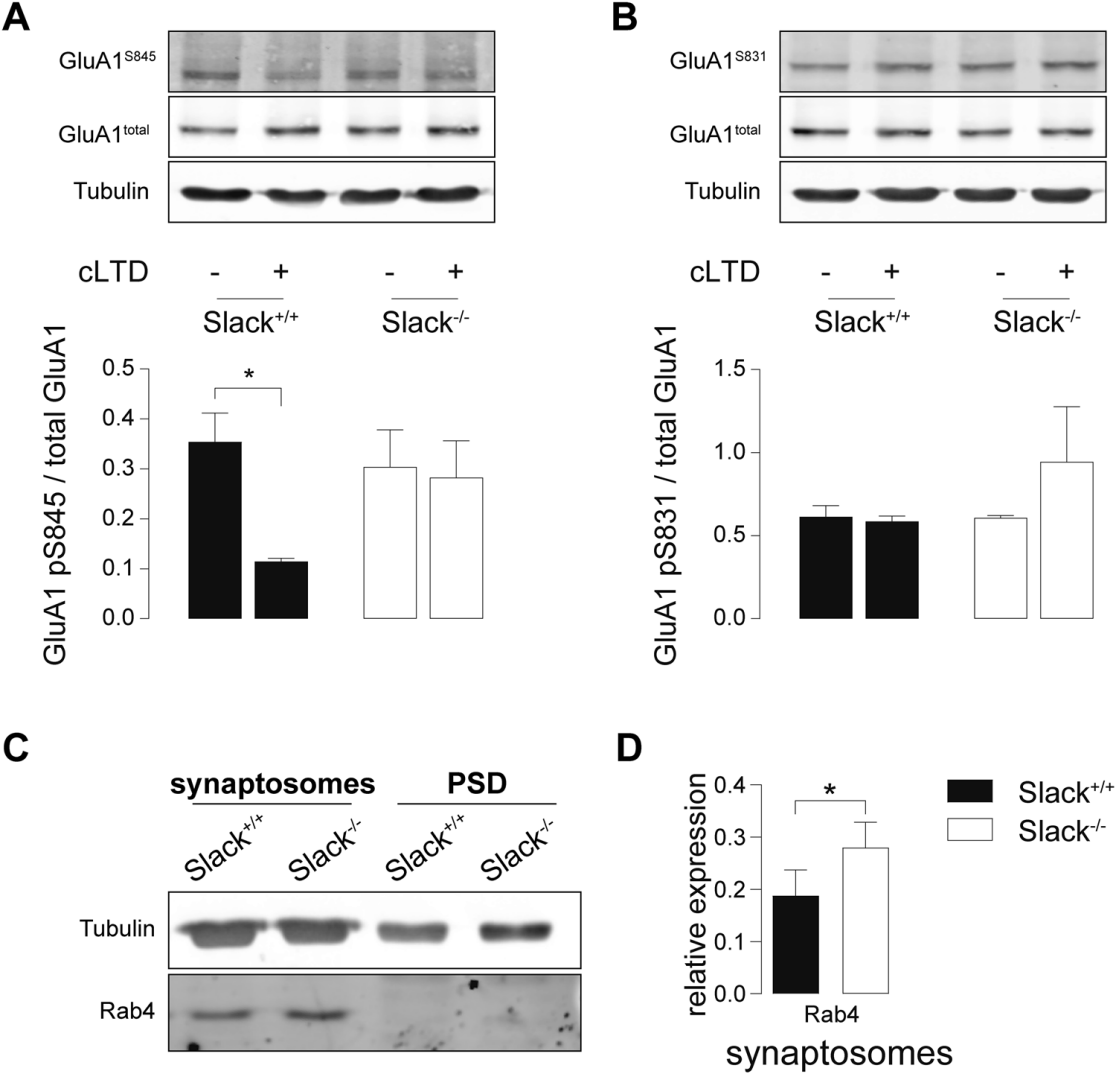
Impaired dephosphorylation of GluA1 S845 after cLTD and increased abundance of Rab4 in *Slack*^*−/−*^. **A**, **B** Hippocampal slices from 4-week-old animals treated with 100 µM NMDA for 5 min and lysed 10 min later for immunodetection of total GluA1 and phosphorylation at residues S845 and S831. cLTD significantly reduced S845 phosphorylation in *Slack*^+*/*+^ but not *Slack*^*−/−*^ (**A**), whereas S831 phosphorylation was unaffected in both genotypes (**B**). Total GluA1 and α-tubulin signals served as control. Top: Representative immunoblots. Bottom: Densitometric quantification of *n* = 3 samples per genotype and condition. **C** Representative Rab4 immunoblot from P9 *Slack*^+*/*+^ and *Slack*^*−/−*^ forebrains for synaptosomal and PSD enriched fractions. Loading control: α-tubulin. **D** Quantification of Rab4 in synaptosomal enriched fractions normalized to α-tubulin (*n* = 5 samples per genotype). Statistics: Two-way ANOVA with Sidak's multiple comparison test (**A**, **B**), Student's t test (**D**). All bar diagrams presented as means ± SEM. See also Table S4

Induction of LTD by appropriate NMDAR stimulation initiates a signaling cascade ultimately dephosphorylating GluA1 at S845, which is a crucial step to allow LTD expression by AMPAR endocytosis [43, 59]. We tested this key mechanism in *Slack*^*−/−*^ after chemical LTD (cLTD), a model of NMDAR-dependent LTD akin to electrically induced LTD particularly in respect to GluA1 phosphorylation states [60]. Compared to vehicle-treated controls, cLTD induction by NMDA application (100 µM, 5 min) significantly reduced S845 phosphorylation in hippocampal slices of 4-weeks-old *Slack*^+*/*+^ but not *Slack*^*−/−*^ (Fig. 4A). Phosphorylation levels of GluA1 S831, which is not associated with LTD [23], were not influenced in both genotypes (Fig. 4B). Despite reduced GluA1 mRNA levels in P28 *Slack*^*−/−*^ (Fig. 2B), we could not detect any differences in total GluA1 protein abundance between hippocampal slices obtained from these older *Slack*^+*/*+^ and *Slack*^*−/−*^ mice (Fig. 4A, B), suggesting that Slack contributes to developmental changes in postsynaptic composition and synaptic plasticity. The observed lack of NMDAR-dependent GluA1 S845 dephosphorylation confirms that hippocampi from *Slack*^*−/−*^ suffer from impaired NMDAR-dependent LTD induction.

### Rab4 is increased in *Slack*^*−/−*^ synaptosomes

Slack directly interacts [61] with the mRNA-binding fragile X mental retardation protein (FMRP) deleted in fragile X syndrome (FXS), a common inherited form of ID and autism spectrum disorders [62]. Slack-FMRP complexes contain mRNA of the activity-regulated cytoskeleton-associated protein (Arc) [61]. Both FMRP and Arc perform vital functions in synaptic plasticity [63, 64]. Expression of either FMRP or Arc, however, was unaltered in synaptosomal or PSD enriched fractions of *Slack*^*−/−*^ (Figures S3A, B). Comparative LC/MS–MS analysis of synaptosomal and PSD enriched biochemical fractions isolated from P9 *Slack*^+*/*+^ and *Slack*^*−/−*^ forebrains identified a number of potentially dysregulated proteins (Figure S3C). Because of their known function in synaptic plasticity [65–69], we further characterized protein phosphatase 1 regulatory subunit 1a (Ppp1r1a), Rab11, RAS protein activator like 1 (Rasal1), Ephrin B3 (Efnb3), and Rab3b by immunoblot (Figure S3D), but were unable to confirm altered expression of any of these proteins by immunoblot in *Slack*^+*/*+^ and *Slack*^*−/−*^ total forebrain (Figure S3E). Beyond that, our proteomic scan identified a high number of vesicle-transport related proteins dysregulated in the absence of Slack (Figure S3C). Therefore, we additionally assessed expression of Rab4, a small GTPase mediating rapid recycling of AMPAR from early endosomes back to the plasma membrane [70] (Fig. 4C). LC/MS–MS identified Rab4 in synaptosomal but not PSD enriched fractions, where its expression was slightly and not significantly higher in *Slack*^*−/−*^ than *Slack*^+*/*+^. Accordingly, Rab4 could not be detected by Western blot in PSD-enriched fractions, but it was significantly upregulated in synaptosomal enriched factions of *Slack*^*−/−*^ (Fig. 4D). An increase in Rab4-mediated recycling of acutely endocytosed AMPAR may serve as one explanation for impaired expression of NMDAR- and particularly mGluR-dependent LTD in *Slack*^*−/−*^ (Figure S5).

### NMDAR-dependent LTD but not LTP is impaired in adult *Slack*^*−/−*^

**Fig. 5:**
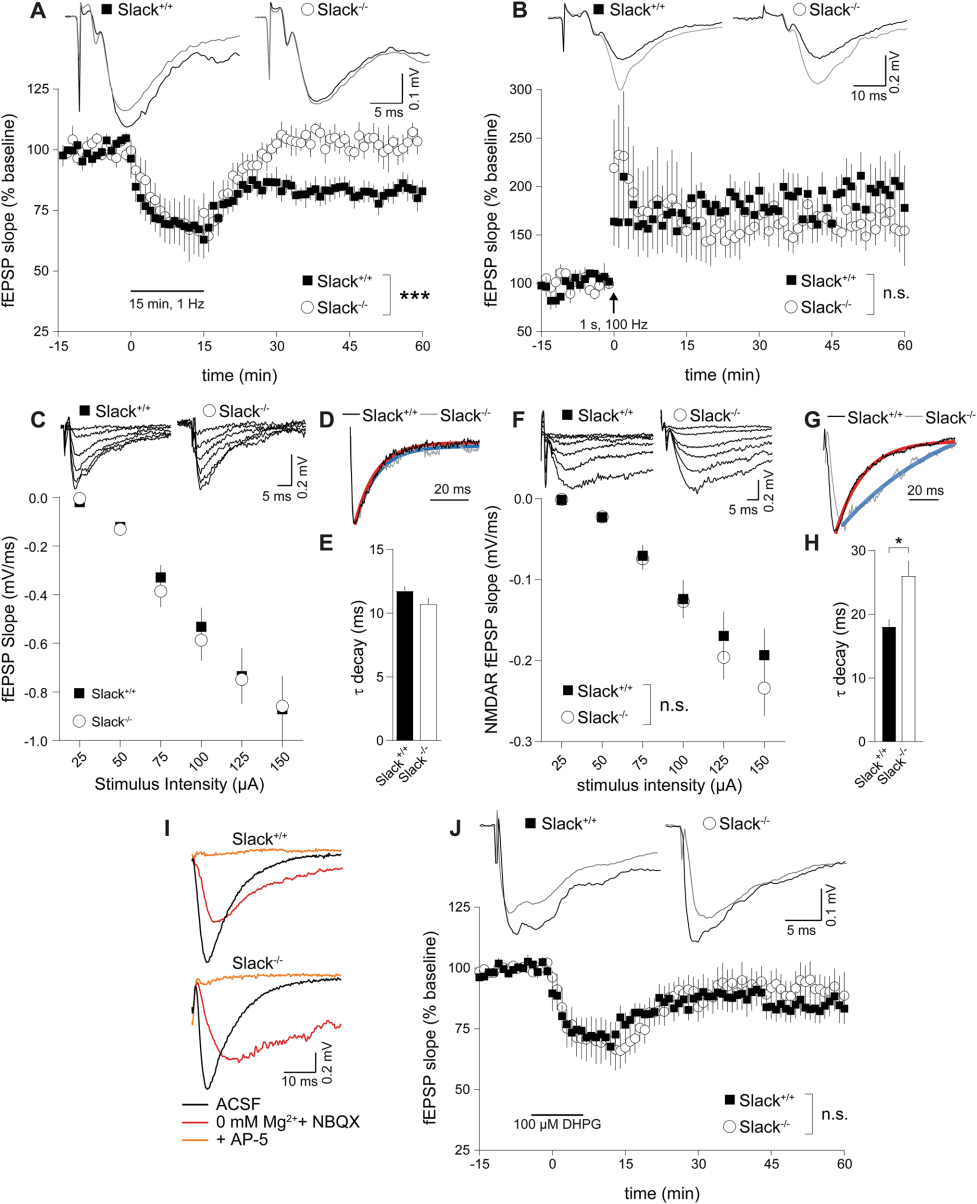
Adult *Slack*^*−/−*^ lack NMDAR-dependent LTD induction but show normal LTP and mGluR-dependent LTD. Initial slopes of Schaffer-collateral fEPSP were recorded from acute forebrain slices of 8- to 12-week-old *Slack*^+*/*+^ and *Slack*^*−/−*^. **A**, **B** 15 min/1 Hz LFS in the presence of 50 µM PiTX caused significant LTD in *Slack*^+*/*+^ (n slices/animals = 6/3), but not in *Slack*^*−/−*^ (*n* = 7/3) (**A**), while a single 100 Hz/1 s HFS elicited significant LTP of comparable strength in both *Slack*^+*/*+^ (*n* = 6/4) and *Slack*^*−/−*^ (*n* = 10/3) (**B**). Top: Sample traces before (black) and after (gray) LTD or LTP induction. **C** Averaged initial fEPSP slopes recorded at stimulation intensities of 25–150 µA in 25 µA increments were not different between *Slack*^+*/*+^ (*n* = 18/9) and *Slack*^*−/−*^ (*n* = 14/3). Top: Representative traces. **D**, **E** Decay time constants assessed by single exponential decay fit of fEPSP responses to 150 µA stimulation was not different between *Slack*^+*/*+^ (*n* = 14) and *Slack*^*−/−*^ (*n* = 10). **D** Representative fEPSP in response to 150 µA stimulation, normalized to peak. Shape of single exponential decay fit function is indicated in red for *Slack*^+*/*+^ and in blue for *Slack*^*−/−*^*.* **E** τ_decay_ from traces elicited by 150 µA stimulation. **F** Initial slopes of NMDAR-mediated fEPSP, isolated in Mg^2+^-free ACSF containing the AMPAR antagonist NBQX (10 µM), recorded at stimulation intensities of 25–150 µA in 25 µA increments were not different between *Slack*^+*/*+^ (*n* = 17/9) and *Slack*^*−/−*^ (*n* = 16/4). Top: Representative traces. **G**–**I** Single exponential decay fit of responses to 150 µA stimulation revealed significantly slower decay time constants of NMDAR fEPSP in *Slack*^*−/−*^ (*n* = 10) compared to *Slack*^+*/*+^ (*n* = 8). **G** Representative NMDAR fEPSP in response to 150 µA stimulation (in Mg^2+^-free ACSF plus NBQX), normalized to peak. Shape of single exponential decay fit function is indicated in red for *Slack*^+*/*+^ and in blue for *Slack*^*−/−*^. **H** τ_decay_ from NMDAR fEPSP traces elicited by 150 µA stimulation. **I** Representative fEPSP in normal ACSF (black, corresponds to traces in **C**), Mg^2+^-free ACSF with NBQX (red, corresponds to traces in **F**) and after addition of the NMDAR antagonist AP-5 (100 µM, orange). **J** Superfusion of 100 µM DHPG for 10 min induced significant mGluR-dependent LTD in *Slack*^+*/*+^ (*n* = 7/4), and *Slack*^*−/−*^ (*n* = 9/3). Top: Representative traces before (black) and after (gray) LTD induction by DHPG. Statistics: Two-way ANOVA with Sidak's multiple comparison test (**A**, **B**, **C**, **F**), Student's t test (**E**). All bar diagrams presented as means ± SEM. See also Table S5

To reconcile the present finding of reduced LTD and LTP in infant *Slack*^*−/−*^ with previously reported normal MWM acquisition and mildly impaired reversal learning in adult *Slack*^*−/−*^ [5], we tested Schaffer-collateral plasticity in 8- to 12-week-old adult mice. Mature *Slack*^*−/−*^ did not overcome deficits in LTD induction, as fEPSP initial slopes in *Slack*^+*/*+^ but not *Slack*^*−/−*^ were significantly depressed in response to the previously established LFS protocol (Fig. 5A). In contrast to infants, we observed significant LTP in adults of both genotypes (Fig. 5B). These LTD and LTP analyses are in accordance with decreased reversal learning and normal MWM acquisition performance of *Slack*^*−/−*^ [5]. In contrast to infant *Slack*^*−/−*^, basal pre- and postsynaptic function of hippocampal synapses including decay kinetics of AMPAR-mediated fEPSP was normal in adult *Slack*^*−/−*^ (Figs. 5C–E and S4). Further, unlike in infant *Slack*^*−/−*^, signal strength in relation to stimulus intensity of NMDAR-mediated Schaffer-collateral fEPSP was normal in adult *Slack*^*−/−*^ (Fig. 5F). Similar to infant *Slack*^*−/−*^, however, NMDAR fEPSP decay kinetics as assessed by single exponential decay fit (compare Figs. 1G and 5G) were significantly slower in adult *Slack*^*−/−*^ than *Slack*^+*/*+^ (compare Figs. 1H and 5H). This points to altered NMDAR-mediated signaling as possible source of persistently impaired LTD induction mechanisms. LTD expression mechanisms, however, seem to function regularly in adult *Slack*^*−/−*^, as mGluR agonist-induced LTD is normal in adult *Slack*^*−/−*^ (Fig. 5J).

**Fig. 6:**
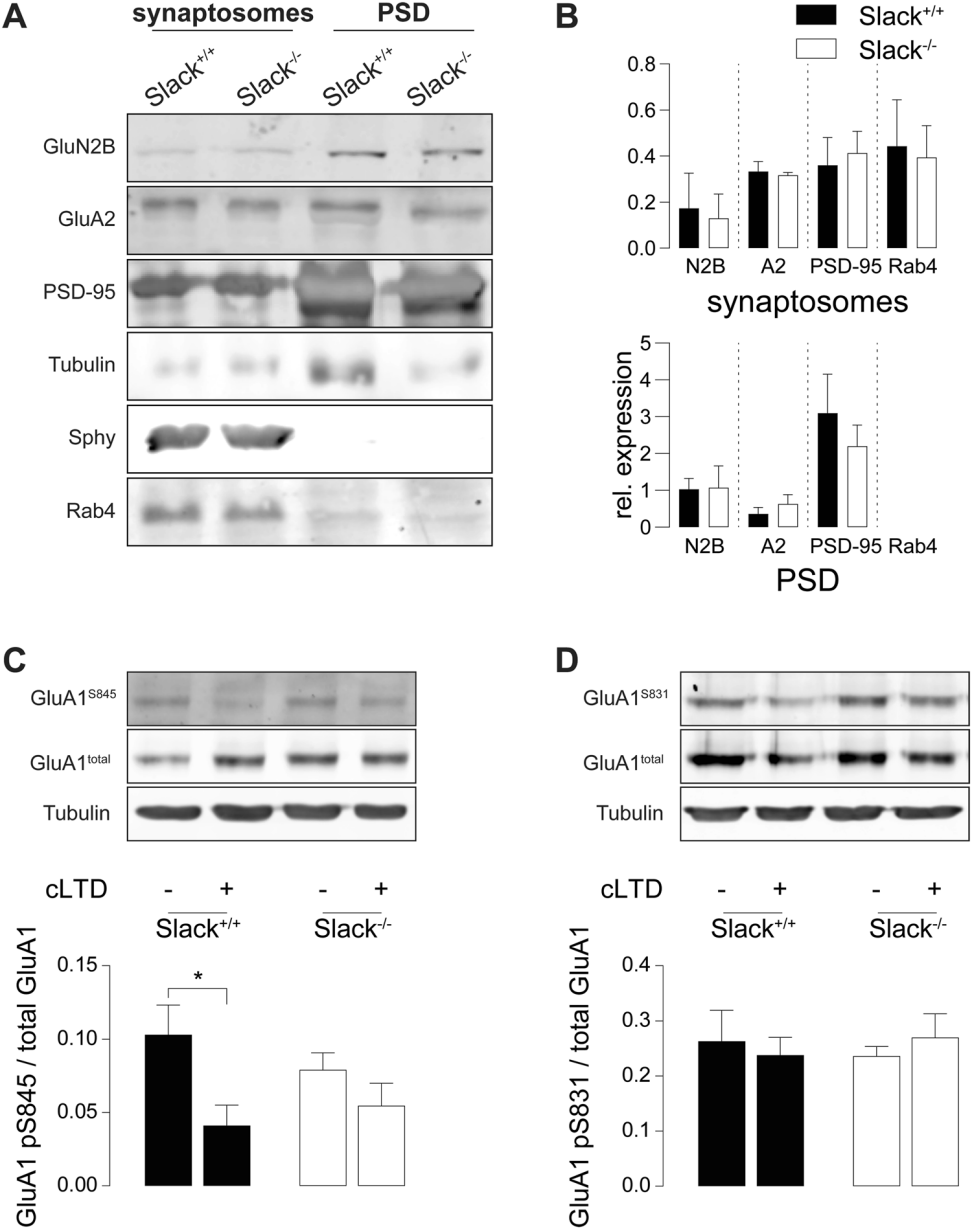
Normal GluN2B expression but impaired dephosphorylation of GluA1 S845 after cLTD in adult *Slack*^*−/−*^*.* **A** Representative immunoblots of biochemical fractions derived from 8- to 12-week-old *Slack*^+*/*+^ and *Slack*^*−/−*^ hippocampi. Separated synaptosomal and PSD enriched fractions were probed with antibodies for GluN2B, GluA2 and Rab4. Controlling for successful fractionation, PSD-95 was enriched in PSD and de-enriched in synaptosomes. Pre-synaptic synaptophysin (Sphy), is absent from PSD but enriched in synaptosomes. **B** Quantification of GluN2B, GluA2, PSD-95 and Rab4 protein band intensities normalized to α-tubulin in synaptosomal (top) and PSD (bottom) enriched fractions did not reveal any differences between genotypes. Each value corresponds to at least *n* = 3 independent preparations of 4–8 pooled hippocampi. Rab4 was not detectable in PSD. **C**, **D** Hippocampal slices from 8-week-old animals treated with 100 µM NMDA for 5 min and lysed 10 min later. cLTD significantly reduced GluA1 S845 phosphorylation in *Slack*^+*/*+^, but not *Slack*^*−/−*^ (**C**) while GluA1 S831 phosphorylation is unaltered in both genotypes (**D**). Total GluA1 levels and α-tubulin signals served as control. Top: Representative immunoblots. Bottom: Densitometric quantification of *n* = 4 samples per genotype and condition. Statistics: Student's t test (**B**), Two-way ANOVA with Sidak's multiple comparison test (**C**, **D**). All bar diagrams presented as means ± SEM. See also Table S6

To explain partially restored synaptic plasticity in adult *Slack*^*−/−*^, we analyzed total synaptosomes and postsynaptic density (PSD) enriched protein fractions from adult *Slack*^+*/*+^ and *Slack*^*−/−*^ hippocampi (Fig. 6A). In contrast to infants, expression of GluN2B and PSD-95 in PSD as well as Rab4 in synaptosomes in adults was not different between *Slack*^+*/*+^ and *Slack*^*−/−*^ (Fig. 6B). Normalization in postsynaptic GluN2B function might sufficiently explain normalization of NMDAR-dependent LTP in adult *Slack*^*−/−*^. Normal Rab4 expression on the other hand could restore LTD expression through AMPAR exocytosis and thus normalize mGluR-dependent LTD. Still, induction of NMDAR-mediated LTD remains impaired in adult *Slack*^*−/−*^, which is confirmed by a significant lack of S845, but not of S831, dephosphorylation at GluA1 after cLTD in adult *Slack*^*−/−*^ (Fig. 6C, D).

## Discussion

Here we demonstrate that infant *Slack*^*−/−*^ hippocampi lack Schaffer-collateral synaptic plasticity in the form of NMDAR-dependent LTP and LTD as well as mGluR-dependent LTD (Fig. 1A, B, J). There are two possible explanations for this unique lack of several forms of hippocampal synaptic plasticity. First, the lack of an important hyperpolarizing conductance in the postsynaptic membrane might cause delayed EPSP decay in vitro (Fig. 1E, H) and thereby altered timing of plasticity-relevant NMDAR-mediated Ca^2+^ signals in vivo. Second, infant *Slack*^*−/−*^ display reduced postsynaptic levels of the NMDAR subunit GluN2B (Figs. 2A, D and 3C). Both observations explain altered NMDAR-dependent synaptic transmission (Fig. 1F–H), which most likely also causes the observed lack of effective dephosphorylation of S845 on the GluA1 subunit of AMPAR by, for instance, protein phosphatase 1 (PP1) in response to cLTD induction [43] that is essential for AMPAR endocytosis (Figs. 4A and S5). Interestingly, the Slack-interacting protein Phactr1 was shown to recruit PP1 to phosphoprotein substrates [4]. Altered localization of Phactr1 due to lack of Slack might alternatively explain lacking GluA1 dephosphorylation in Slack^−/−^ (Fig. 6C). At the same time, AMPAR exocytosis may be facilitated by upregulation of the small GTPase Rab4 in *Slack*^*−/−*^ to interfere with LTD expression (Figure S5). It is presently not clear if excessive recycling of AMPAR-containing early endosomes occurs independent of NMDAR signaling (Fig. 4D) or stems from additional Rab4 actions on NMDAR currents [71]. It is also tempting to speculate that the increased Rab4 expression represents a homeostatic response to accelerate AMPAR recycling and AMPAR-mediated Na^+^ influx, which reportedly activates Slack currents in spinal cord neurons [72]. Moreover, increased Rab4 activity might explain why mGluR-dependent LTD is also defective in *Slack*^*−/−*^, as this form of LTD does not depend on NMDAR activity. Interestingly, GluN2B and Rab4 expression (Fig. 6B) were normal in adult *Slack*^*−/−*^, possibly explaining restored LTP as well as mGluR-dependent LTD (Fig. 5B, J). NMDAR-dependent LTD, however, remains compromised even in adult *Slack*^*−/−*^ (Fig. 5A), which is in accordance with earlier findings of normal memory acquisition but impaired cognitive flexibility in *Slack*^*−/−*^ mice performing the MWM [5]. So far, however, our data does not give a definitive answer how the observed phenotype of impaired synaptic plasticity is caused by ablation of Slack.

ID in patients suffering from Slack-associated epilepsies is most likely not due to seizure activity but Slack dysfunction, as mutations of unrelated but also highly disease-relevant proteins can cause similar seizure pathologies that present without ID [6]. The present data strongly suggest that altered Slack function in these patients leads to impaired synaptic plasticity, which is the underlying issue in the development of ID [24]. Interestingly, epilepsy syndromes associated with Slack mutations may derive from channel proteins that exhibit either increased or decreased currents [8, 9], whereas both types of mutations are linked with ID [10, 11, 20, 73] and mice carrying a gain-of-function mutation show impaired procedural learning [20]. Because the herein studied model completely lacks functional Slack proteins (Figs. 2C and S2), we conclude that any disbalance in channel levels and thus Slack activity may impact proper synaptic functions, but cannot exclude that gain-of- and loss-of-function mutations promote ID through completely different mechanisms. Additionally, *Slack*^*−/−*^ display altered NMDAR-mediated signaling and reduced postsynaptic GluN2B protein levels (Figs. 1F–H, 2D and 3C). So far, several mutations in NMDAR subunits, also in GluN2B, were associated with the development of ID [74]. Overall, our results strongly suggest that NMDAR signaling and NMDAR-mediated synaptic plasticity are promising therapeutic targets to treat ID associated with Slack mutations. This is additionally encouraged by recent data suggesting that developmental impacts of NMDAR dysfunction could be ameliorated by therapeutic interventions in adulthood [75].

There are several conceivable possibilities how altered Slack function could influence synaptic plasticity. Slack contributes to neuronal resting membrane potential and excitability levels [19]. They are functionally coupled to AMPAR as local AMPAR-mediated Na^+^ entry during action potentials (AP) can activate Slack [72, 76], and we indeed find that Slack expression feeds back to AMPAR endocytosis (Figs. 5, 6C, D and S5). So far, functional coupling of Slack to NMDAR was only demonstrated to modulate excitotoxic brain damage and neuronal cell death [77]. A molecular link is conceivable, as Slack interacts with PSD-95 [78], which in turn binds to GluN2 subunits of NMDAR [54]. Through this functional coupling between Slack and NMDAR, Slack-mediated hyperpolarization, after a physiological level of NMDAR activation, could hyperpolarize the membrane, stabilize the Mg^2+^ block of the NMDAR and subsequently reduce Ca^2+^ entry through NMDAR to reduce neuronal excitability. Slack knockout would thus lead to membrane depolarization and increased NMDAR-mediated Ca^2+^ signaling. As LTD induction is not permitted at increased Ca^2+^ levels [43], this could explain the observed lack of LTD (Fig. 1A). It is, however, harder to reconcile with reduced LTP (Fig. 1B), which is usually increased by potassium channel deletion [79] and elevated Ca^2+^ levels. Furthermore, we did not detect different glutamate-induced Ca^2+^ influx between *Slack*^+*/*+^ and *Slack*^*−/−*^ (Fig. 3B). An alternative explanation is that lack of Slack-mediated slow afterhyperpolarization (sAHP) following AP prevents repolarization and thus reduces the achievable AP firing rate of the postsynaptic neurons. Such a mechanism might manifest as delayed fEPSP decay (Figs. 1E, H, 5E, H). A similar mechanism was previously described in cerebellar Purkinje cells of BK knockout mice [80] and might explain the lack of reduced LTP and maybe also LTD. Vice versa, NMDAR-mediated Na^+^ signals [54] may also lead to Slack activation which could contribute to inhibitory fine tuning of NMDAR activity. Altered NMDA-mediated signaling (Figs. 1G, H and 3C) through aberrant Ca^2+^ signals caused by increased or decreased Slack activity might thus disturb optimal synaptic connectivity in terms of synaptic plasticity as well as learning and memory.

The simultaneous lack of LTD and LTP in infant *Slack*^*−/−*^, which both critically depend on NMDAR-mediated signaling [21], might be explained by reduced levels of the NMDAR subunit GluN2B, the predominant hippocampal GluN2 subunit in early development that confers increased Ca^2+^ permeability to NMDAR [54]. Indeed, lack of GluN2B was previously associated with both impaired LTD and LTP ([55, 56], but also see [57]). Decreased NMDAR-mediated Ca^2+^ entry leads to reduced phosphatase activation which precludes S845 dephosphorylation and consequently AMPAR endocytosis [43]. As NMDAR-mediated Ca^2+^ entry was not different between *Slack*^+*/*+^ and *Slack*^*−/−*^, however, the mechanism underlying reduced GluN2B expression in *Slack*^*−/−*^ remains unknown. The concomitantly impaired NMDAR-independent mGluR-dependent LTD (Fig. 1J) indicates a defective LTD expression mechanism. Indeed, increased Rab4 levels (Fig. 4D) could lead to increased recycling of AMPAR from early endosomes back to the plasma membrane (Figure S5), a mechanisms that would in turn prevent effective removal of AMPAR from the surface andthus LTD [70].

An interesting observation is that LTP, mGluR LTD, but not NMDAR-LTD is restored in adults (Fig. 5). For one, this could be due to developmental switches in the signaling cascades necessary for LTP induction [81] around P9 in mice or mGluR LTD induction [82] around P15 in rats. Alternatively, normalized expression of GluN2B and Rab4 (Fig. 6B) might account for re-established NMDAR-dependent LTP and mGluR LTD, respectively, while the persisting impairment of NMDAR-dependent LTD could be explained by the remaining slight perturbation of NMDAR signaling as manifested in slower decay of NMDAR-fEPSP in adults (Fig. 5H). The persistence of reduced NMDAR-dependent LTD confirms our previous findings that adult *Slack*^*−/−*^ show normal memory acquisition, but impaired reversal in the MWM. Interestingly, compared to *Slack*^+*/*+^, adult *Slack*^*−/−*^ tend to use a serial and not spatial search strategy in the Barnes maze [5]. This points to persistent changes in *Slack*^*−/−*^ memory performance that hint to developmental differences in spatial learning strategies between *Slack*^+*/*+^ and *Slack*^*−/−*^, possibly due to altered synaptic plasticity in early life. Apparently, the ability of the maturating brain to compensate for improper hippocampal Slack activit y during development is rather limited for the expression of NMDAR-dependent LTD, which would explain the observed behavioral phenotypes of *Slack*^*−/−*^ mice, if we at least accept a causal link between in vitro recorded LTD and distinct learning performances in vivo*.*

## Supplementary Information

Below is the link to the electronic supplementary material.

Supplement ary fil e1 (PDF 1194 KB)

## Data Availability

All data generated or analyzed during this study that are not included in this published article and its supplementary information files are available from the corresponding authors on reasonable request.
