# Supplementary material for: The Na+-activated K+ channel Slack contributes to synaptic development and plasticity
Source: Cell Mol Life Sci. 2021 Oct 18;78(23):7569–87. doi: 10.1007/s00018-021-03953-0 (PMC8629810; doi:10.1007/s00018-021-03953-0)
Supplement: Supplementary file 1 — Supplement ary fil e1 (PDF 1194 KB) [file 18_2021_3953_MOESM1_ESM.pdf]

Supplemental materials for the manuscript

The Na^+-activated K^+ channel Slack contributes to synaptic development and plasticity

Lucas Matt[1,*], Thomas Pham[1], David Skrabak[1], Felix Hoffmann[1,2], Philipp Eckert[1], Jiaqi Yin[1], Miriam Gisevius[1], Rebekka Ehinger[1], Anne Bausch[1], Marius Ueffing[2], Karsten Boldt[2], Peter Ruth[1], Robert Lukowski[1,*]

[1] Department of Pharmacology, Toxicology and Clinical Pharmacy, Institute of Pharmacy, University of Tübingen, 72076 Tübingen, Germany

[2] Institute for Ophthalmic Research, Molecular Biology of Retinal Degenerations and Medical Bioanalysis, University of Tübingen, 72076 Tübingen, Germany

*Co-corresponding authors: Lucas Matt, email: lucas.matt@uni-tuebingen.de; Robert Lukowski, email: robert.lukowski@uni-tuebingen.de.

Abbreviated title: Slack in synaptic plasticity

SUPPLEMENTAL TABLES

Table S1. Values and statistics for Figure 1.

panel	genotype	baseline (%-baseline)	stimulated (%-baseline	n (slice/animal)	Statistics	p
A	Slack$^{+/+}$	100.2 ± 0.2	68.9 ± 0.2	10/5	$F_{3,36}$ = 12.3	<0.001 vs. baseline
	Slack$^{-/-}$	99.8 ± 0.2	96.9 ± 7.3	10/4		<0.001 vs. Slack$^{+/+}$
B	Slack$^{+/+}$	99.7 ± 0.7	159.4 ± 11.9	13/8	$F_{3,40}$ = 17.2	<0.001 vs. baseline
	Slack$^{-/-}$	99.9 ± 0.3	107.5 ± 4.6	9/4		<0.001 vs. Slack$^{+/+}$

panel	genotype	stimulus intensity (µA)	Slope (mV/ms)	n (slice/animal)	Statistics	p
C	Slack$^{+/+}$	25	-0.037 ± 0.003	12/4	n.s.	
		50	-0.047 ± 0.004			
		75	-0.105 ± 0.013			
		100	-0.167 ± 0.023			
		125	-0.193 ± 0.025			
		150	-0.215 ± 0.025			
	Slack$^{-/-}$	25	-0.035 ± 0.008	7/3		
		50	-0.041 ± 0.003			
		75	-0.088 ± 0.012			
		100	-0.135 ± 0.024			
		125	-0.150 ± 0.030			
		150	-0.175 ± 0.037			

panel	genotype	τ decay (ms)	n	statistics	p
E	Slack$^{+/+}$	6.8 ± 0.6	12	unpaired t-test	
	Slack$^{-/-}$	10.1 ± 0.9	8		<0.01 vs. Slack$^{+/+}$

panel	genotype	stimulus intensity (µA)	Slope (mV/ms)	n (slice/animal)	Statistics	p
F	Slack$^{+/+}$	25	-0.032 ± 0.004	18/3	$F_{1,150}$ = 23.2, $p < 0.001$	
		50	-0.064 ± 0.010			
		75	-0.118 ± 0.017			
		100	-0.151 ± 0.023			
		125	-0.178 ± 0.026			
		150	-0.206 ± 0.031			
	Slack$^{-/-}$	25	-0.032 ± 0.004	9/2		
		50	-0.04 ± 0.005			
		75	-0.058 ± 0.008			
		100	-0.074 ± 0.013			
		125	-0.084 ± 0.015			0.02 vs. Slack$^{+/+}$
		150	-0.086 ± 0.017			0.001 vs Slack$^{+/+}$

panel	genotype	τ decay (ms)	n	statistics	p
H	Slack$^{+/+}$	9.5 ± 1.3	18	unpaired t-test	
	Slack$^{-/-}$	39.1 ± 6.6	9		<0.001 vs. Slack$^{+/+}$

panel	genotype	baseline (%-baseline)	stimulated (%-baseline	n (slice/animal)	Statistics	p
J	Slack$^{+/+}$	99.9 ± 1.0	63.4 ± 4.7	8/2	$F_{3,32}$ = 21.09, $p < 0.001$	<0.001 vs. baseline
	Slack$^{-/-}$	99.5 ± 0.5	92.2 ± 5.2	10/3		<0.001 vs. Slack$^{+/+}$

Table S2. Values and statistics for Figure 2.

panel	protein	n	statistics	age (d)	p
A	GluN1	3-4	$F_{(1,28)} = 3.5$		
	GluN2A	3-4	$F_{(1,29)} = 1.9$	14	0.04
	GluN2B	3-4	$F_{(1,26)} = 90.3$, $p < 0.001$	1	<0.001
				7	<0.001
				14	<0.001
	GluN3A	5-8	$F_{(1,42)} = 1.7$	1	<0.001
B	GluA1	3	$F_{(1,19)} = 52.6$, $p < 0.001$	21	<0.001
				28	0.003
	GluA2	3	$F_{(1,18)} = 0.26$		
	GluA3	3	$F_{(1,19)} = 6.7$, $p = 0.02$	21	<0.001
	GluA4	3	$F_{(1,19)} = 1.3$		

panel	fraction	protein	genotype	relative expression	n	p
D	synaptosomes	GluN1	Slack$^{+/+}$	0.070 ± 0.026	3	
			Slack$^{-/-}$	0.043 ± 0.018		
		GluN2A	Slack$^{+/+}$	0.013 ± 0.003	3	
			Slack$^{-/-}$	0.016 ± 0.006		
		GluN2B	Slack$^{+/+}$	0.128 ± 0.050	11	
			Slack$^{-/-}$	0.095 ± 0.029		
		GluN3A	Slack$^{+/+}$	0.054 ± 0.010	5	
			Slack$^{-/-}$	0.037 ± 0.011		
	PSD	GluN1	Slack$^{+/+}$	0.716 ± 0.327	3	
			Slack$^{-/-}$	0.496 ± 0.302		
		GluN2A	Slack$^{+/+}$	N.A.	N.A.	
			Slack$^{-/-}$	N.A.		
		GluN2B	Slack$^{+/+}$	1.079 ± 0.281	8	
			Slack$^{-/-}$	0.675 ± 0.176		0.017 vs. Slack$^{+/+}$
		GluN3A	Slack$^{+/+}$	0.129 ± 0.042	5	
			Slack$^{-/-}$	0.097 ± 0.012		

panel	fraction	protein	genotype	relative expression	n	p
E	synaptosomes	GluA1	Slack$^{+/+}$	0.192 ± 0.052	3	
			Slack$^{-/-}$	0.109 ± 0.017		
		GluA2	Slack$^{+/+}$	1.027 ± 0.688	3	
			Slack$^{-/-}$	0.716 ± 0.348		
		PSD-95	Slack$^{+/+}$	0.36 ± 0.138	7	
			Slack$^{-/-}$	0.335 ± 0.13		
	PSD	GluA1	Slack$^{+/+}$	0.329 ± 0.063	3	
			Slack$^{-/-}$	0.145 ± 0.010		
		GluA2	Slack$^{+/+}$	3.747 ± 0.758	3	
			Slack$^{-/-}$	4.887 ± 1.488		
		PSD-95	Slack$^{+/+}$	0.998 ± 0.268	7	
			Slack$^{-/-}$	2.051 ± 0.664		0.046 vs Slack$^{+/+}$

Table S3. Values and statistics for Figure 3.

panel C	genotype	stim	AUC (R/R0 x s)	n (recordings/cultures/cells)	p
	Slack$^{+/+}$	**1st**	0.325 ± 0.027	6/2/90	
		**2nd**	0.093 ± 0.012		
	Slack$^{-/-}$	**1st**	0.511 ± 0.095	6/3/96	0.097 vs Slack$^{+/+}$
		**2nd**	0.259 ± 0.046		< 0.01 vs Slack$^{+/+}$

Table S4. Values and statistics for Figure 4.

panel	genotype	pS845/GluA1 unstimulated	pS845/GluA1 stimulated	n	statistics	p
A	Slack$^{+/+}$	0.35 ± 0.06	0.11 ± 0.01	3	$F_{1,8}$ = 1.0	0.04 vs. unstim.
	Slack$^{-/-}$	0.30 ± 0.01	0.28 ± 0.07			

panel	genotype	pS831/GluA1 unstimulated	pS831/GluA1 stimulated	n	statistics	p
B	Slack$^{+/+}$	0.61 ± 0.07	0.59 ± 0.03	3	$F_{1,8}$ = 1.1	
	Slack$^{-/-}$	0.61 ± 0.02	0.94 ± 0.33			

panel	fraction	protein	genotype	relative expression	n	p
D	synaptosomes	Rab4	Slack$^{+/+}$	0.187 ± 0.049	5	
			Slack$^{-/-}$	0.279 ± 0.048		0.029 vs. Slack$^{+/+}$

Table S5. Values and statistics for Figure 5.

panel	genotype	baseline (%-baseline)	stimulated (%-baseline	n (slice/animal)	statistics	p
A	Slack$^{+/+}$	100.3 ± 0.3	82.5 ± 3.9	6/3	$F_{3,22} = 11.0$	< 0.001 vs. baseline
	Slack$^{-/-}$	100 ± 0.02	104.7 ± 2.7	7/3		< 0.001 vs. Slack+/+

panel	genotype	baseline	stimulated	n	statistics	p
B	Slack$^{+/+}$	99.6 ± 1.5	184.6 ± 22.6	6/4	$F_{3,28} = 8.1$	< 0.01 vs. baseline
	Slack$^{-/-}$	100.0 ± 0.1	162.1 ± 16.2	10/3		< 0.01 vs. baseline

panel	genotype	stimulus intensity (µA)	Slope (mV/ms)	n (slice/animal)	statistics	p
C	Slack$^{+/+}$	25	-0.02 ± 0.02	18/9		
		50	-0.12 ± 0.02			
		75	-0.33 ± 0.05			
		100	-0.53 ± 0.08			
		125	-0.74 ± 0.12			
		150	-0.87 ± 0.14			
	Slack$^{-/-}$	25	-0.004 ± 0.008	14/3		
		50	-0.13 ± 0.03			
		75	-0.39 ± 0.07			
		100	-0.59 ± 0.08			
		125	-0.75 ± 0.09			
		150	-0.86 ± 0.11			

panel	genotype	τ decay (ms)	n	statistics	p
E	Slack$^{+/+}$	11.8 ± 0.4	14		
	Slack$^{-/-}$	10.7 ± 0.5	10		

panel	genotype	stimulus intensity (µA)	Slope (mV/ms)	n (slice/animal)	statistics	p
F	Slack$^{+/+}$	25	-0.001 ± 0.002	17/9	$F_{1,238} = 8.4$	
		50	-0.023 ± 0.005			
		75	-0.070 ± 0.014			
		100	-0.124 ± 0.024			
		125	-0.169 ± 0.030			
		150	-0.193 ± 0.033			
	Slack$^{-/-}$	25	-0.001 ± 0.002	16/4		
		50	-0.022 ± 0.007			
		75	-0.074 ± 0.013			
		100	-0.127 ± 0.016			
		125	-0.196 ± 0.027			
		150	-0.234 ± 0.034			

panel	genotype	τ decay (ms)	n	statistics	p
H	Slack$^{+/+}$	18.1 ± 1.1	8	unpaired t-test	
	Slack$^{-/-}$	26.1 ± 2.3	10		0.0105 vs. Slack$^{+/+}$

panel	genotype	baseline (%-baseline)	stimulated (%-baseline	n (slice/animal)	Statistics	p
J	Slack$^{+/+}$	99.9 ± 0.2	84.3 ± 4.5	7/4	$F_{3,28} = 3.53$	0.04 vs. baseline
	Slack$^{-/-}$	100.1 ± 0.04	91.1 ± 6.0	9/3	$p = 0.03$	0.26 vs. baseline

Table S6. Values and statistics for Figure 6.

panel	fraction	protein	genotype	relative expression	n	p
B	synaptosomes	GluN2B	Slack$^{+/+}$	0.17 ± 0.15	3	
			Slack$^{-/-}$	0.13 ± 0.11		
		GluA2	Slack$^{+/+}$	0.33 ± 0.043	3	
			Slack$^{-/-}$	0.32 ± 0.012		
		PSD-95	Slack$^{+/+}$	0.36 ± 0.12	3	
			Slack$^{-/-}$	0.41 ± 0.094		
		Rab4	Slack$^{+/+}$	0.44 ± 0.20	5	
			Slack$^{-/-}$	0.39 ± 0.14		
	PSD	GluN2B	Slack$^{+/+}$	1.0 ± 0.30	3	
			Slack$^{-/-}$	1.1 ± 0.59		
		GluA2	Slack$^{+/+}$	0.36 ± 0.17	3	
			Slack$^{-/-}$	0.63 ± 0.25		
		PSD-95	Slack$^{+/+}$	3.1 ± 1.1	3	
			Slack$^{-/-}$	2.2 ± 0.57		
		Rab4	Slack$^{+/+}$	N.A.	3	
			Slack$^{-/-}$	N.A.		

panel	genotype	pS845/GluA1 unstimulated	pS845/GluA1 stimulated	n	statistics	p
C	Slack$^{+/+}$	0.10 ± 0.02	0.04 ± 0.01	4	$F_{1,12}$ = 7.7	0.03 vs. unstim.
	Slack$^{-/-}$	0.08 ± 0.01	0.06 ± 0.02			

panel	genotype	pS831/GluA1 unstimulated	pS831/GluA1 stimulated	n	statistics	p
D	Slack$^{+/+}$	0.26 ± 0.06	0.24 ± 0.03	4	$F_{1,12}$ = 0.004	
	Slack$^{-/-}$	0.24 ± 0.02	0.27 ± 0.04			

Table S7. Oligonucleotides used in this study

genotyping

	primer	sequence 5' to 3'
**Slack**	for	AGGGGCTGAGAGGGGTCTCG
	rev1	TGGGTAGGGCTGCCACAAGC
	rev2	GCCACAATCTGTTCCTTGGCAC

qRT-PCR

**GluA1**	for	TGGTGGTGGTGGACTGTGAA
	rev	GGTTGGCGAGGATGTAGTGG
**GluA2**	for	AGCACTCCTTAGCTTGATTGAGT
	rev	CCACTTCTTCTCCGCAGCAG
**GluA3**	for	AGAACACCACTGAGAAGCCCT
	rev	CCTCTGGAGAACTGGGAGCA
**GluA4**	for	CCCAATGCATCTGAAGCCCC
	rev	TGGCAAACACCCCTCTAGAA
**GluN1**	for	AGGAAGATCATCTGGCCAGGA
	rev	GGGCTTGACATACACGAAGGG
**GluN2A**	for	GAGACCCCGCTACACACTCT
	rev	TCAGCACGATCACCACAAGC
**GluN2B**	for	CGCCCAGATCCTCGATTTCA
	rev	ACTGGAAGAACATGGAGGACTCA
**GluN3A**	for	TGGCTGCTGTCATGGTAGGT
	rev	CACTGCTTTCCCGGACAGTT
**HPRT**	for	CATTATGCCGAGGATTTGGA
	rev	CCTTCATGACATCTCGAGCA
**Slack**	for	CTGCTGTGCCTGGTCTTCA
	rev	AAGGAGGTCAGCAGGTTCAA
**Slick**	for	CTCGCGCTTTCAAAACTGGA
	rev	ACTCTTCCCGCAGCAAAAGG

Table S8. Primary antibody information.

Target	Source	Catalog #	Species / Isotype	Figure
Arc	Synaptic Systems	156 003	rabbit polyclonal	S3
Ephrin-B3	Elabscience	E-AB-31352	rabbit polyclonal	S3
FMRP	Cell Signaling	4317	rabbit polyclonal	S3
GluA1	Origene	TA326534	mouse IgG1	2,4,6
GluA1^{pS845}	Millipore	AB5849	rabbit polyclonal	4,6
GluA1 ^{pS831}	Cell Signaling	75574	rabbit polyclonal	4,6
GluA2	Cell Signaling	13607	rabbit polyclonal	2
GluN1	Origene	TA326536	mouse IgG1	2
GluN2A	Origene	TA326537	mouse IgG2a	2
GluN2B	Cell Signaling	14544	rabbit polyclonal	2
GluN3A	alomone labs	AGC-030	rabbit polyclonal	2
Ppp1r1a	Biorybt	Orb215507	rabbit polyclonal	S3
PSD-95	NeuroMab	75-028	mouse IgG2a	2,S3
Rab3b	cusabio	CSB-PA019195LA01HU	rabbit polyclonal	S3
Rab4	Cell Signaling	2167	rabbit polyclonal	S3
Rab11	Cell Signaling	5589	rabbit polyclonal	S3
Rasal1	Elabscience	E-AB-15329	rabbit polyclonal	S3
Slack	NeuroMab	75-051	mouse IgG1	2,S2
Synaptophysin	Synaptic Systems	101011	mouse IgG1	2,S3
α-Tubulin	Cell Signaling	3873	mouse IgG1	2,4,6,S2,S3

SUPPLEMENTAL FIGURES AND LEGENDS

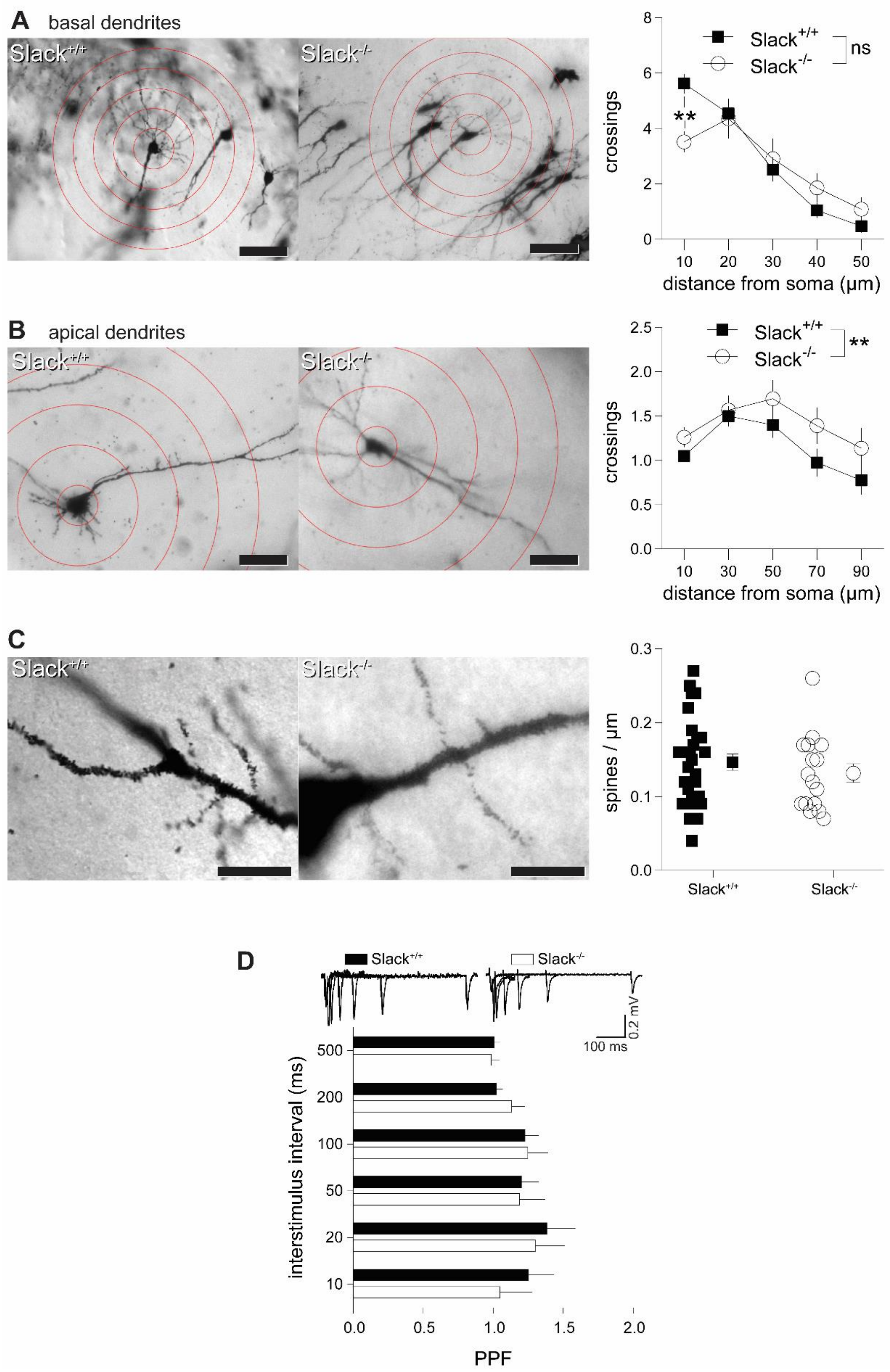

Figure S1. CA1 pyramidal cell morphology and presynaptic function in infant *Slack*$^{-/-}$.

(**A-C**) 150 µm thick forebrain slices from P9 *Slack*$^{+/+}$ and *Slack*$^{-/-}$ mice were stained using the Golgi-technique. Z-stack images were taken using a 20x objective. Representative images are shown on the left, quantification on the right.

(**A**) The number of basal dendrites crossing concentric circles at the indicated distance from the soma was overall not different between *Slack*$^{+/+}$ (n = 40 cells from 4 preparations) and *Slack*$^{-/-}$ (n = 23 cells from 4 preparations) with *Slack*$^{-/-}$ dendrites crossing significantly less only at 10 µm from the soma (two-way ANOVA with Sidak's multiple comparisons test, $F_{1,305} = 0.43$, n.s. for genotype, $p < 0.01$ at 10 µm).

(**B**) The number of apical dendrites crossing concentric circles at the indicated distance from the soma was significantly (two-way ANOVA, $F_{1,305} = 6.8$, $p = 0.0096$) higher for all distances in *Slack*$^{-/-}$ (n = 23 cells from 4 preparations) compared to *Slack*$^{+/+}$ (n = 40 cells from 4 preparations.

(**C**) Spine density on secondary dendrites was determined in Z-stacks taken with a 63x objective. Spine density was not different (unpaired t-test) between *Slack*$^{+/+}$ (0.147 ± 0.011 µm^{-1}, n = 29 images from 4 preparations) and *Slack*$^{-/-}$ (0.132 ± 0.013 µm^{-1}, n = 16 from 4 preparations).

(**D**) Paired-pulse facilitation (PPF) of Schaffer-collateral fEPSP recorded from acute forebrain slices of P6 to P14 *Slack*$^{+/+}$ and *Slack*$^{-/-}$ mice was not different between *Slack*$^{+/+}$ (n = 13 slices from 4 animals) and *Slack*$^{-/-}$ (n = 8 slices from 4 animals) for all inter-stimulus intervals. Traces from representative recordings shown on top (*Slack*$^{+/+}$: 10 ms: 1.2 ± 0.2 mV, 20 ms: 1.4 ± 0.2 mV, 50 ms: 1.2 ± 0.1 mV, 100 ms: 1.2 ± 0.1 mV, 200 ms: 1.02 ± 0.04 mV, 500 ms: 1.01 ± 0.04 mV; *Slack*$^{-/-}$: 10 ms: 1.1 ± 0.2 mV, 20 ms: 1.3 ± 0.20 mV, 50 ms: 1.2 ± 0.2 mV, 100 ms: 1.3 ± 0.2 mV, 200 ms: 1.1 ± 0.1 mV, 500 ms: 1.0 ± 0.1 mV).

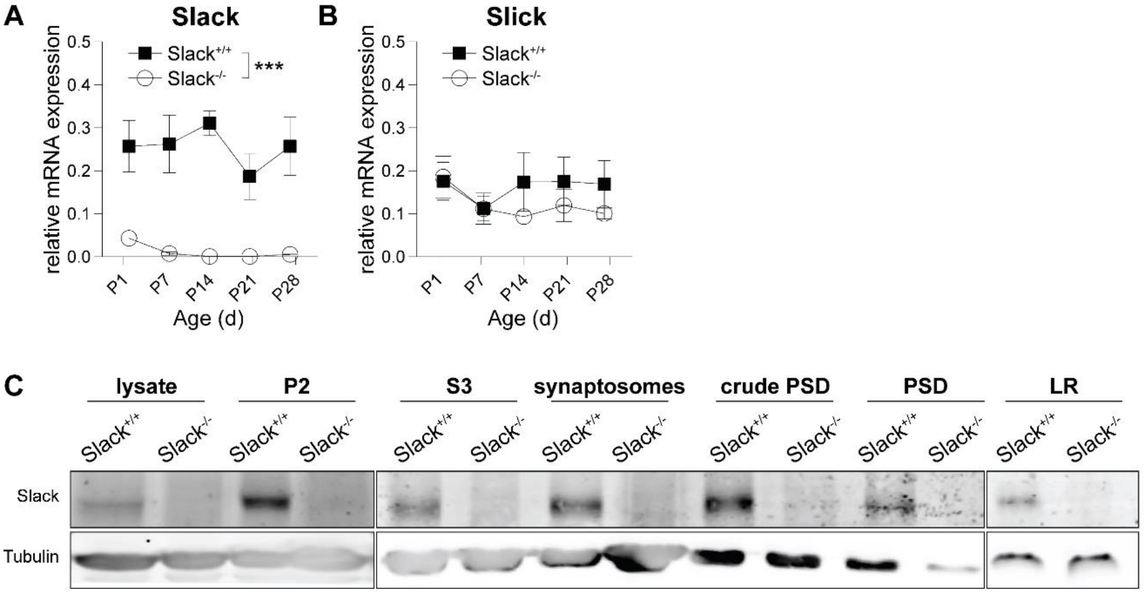

Figure S2. Unaltered Slick transcript levels in infant *Slack*$^{-/-}$ mice and presence of Slack in all biochemical fractions.

(**A-B**) Transcript levels were quantified by RT-PCR of mRNA isolated at weekly intervals during the first moth of life from the hippocampi of *Slack*$^{+/+}$ and *Slack*$^{-/-}$ mice. Results were normalized to HPRT and tested for statistical significance by two-way ANOVA with Sidak's multiple comparison test.

(**A**) Slack transcript levels in *Slack*$^{+/+}$ were steady between P1 and P28. Slack transcripts were not detectable in *Slack*$^{-/-}$ (n = 6, two-way ANOVA, $F_{1,42} = 69.1$, $p < 0.001$).

(**B**) Slick transcript levels in *Slack*$^{+/+}$ and *Slack*$^{-/-}$ did not differ between P1 and P28 (n = 6). Slick transcript levels remained stable during the observed time period.

(**C**) Representative immunoblots of biochemical fractions isolated from *Slack*$^{+/+}$ and *Slack*$^{-/-}$ P9 mouse brain tissue showing expression of Slack in lysate, membrane enriched (P2), vesicular supernatant (S3), synaptosomal, crude PSD, PSD and lipid raft (LR) enriched fractions. Loading controls provided by α-tubulin immunoreactivity.

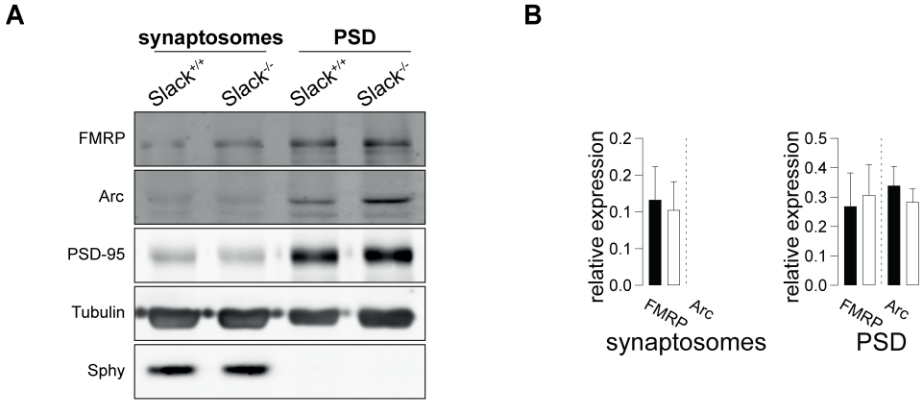

C

	protein ID	gene name	ratio -/-/+/+	signifi-cance B	T-test p	count	cover-age [%]	intens ity
synaptosomes	Q91WK5	Gcsh	-25.43	0.00	0.02	22	18	30
	**Q9ERT9**	**Ppp1r1a**	**-24.77**	**0.00**	**0.03**	**18**	**47**	**29**
	Q8VE70	Pdcd10	-23.93	0.29	0.03	18	22	28
	Q99LG2	Tnpo2	-23.86	0.30	0.03	20	14	28
	P37040	Por	-0.54	0.05	0.04	156	42	32
	Q99PU5	Acsbg1	-0.50	0.06	0.03	168	42	32
	P12849	Prkar1b	-0.46	0.06	0.05	120	40	31
	O70503	Hsd17b12	-0.27	0.33	0.05	115	45	32
	Q8VE33	Gdap1l1	-0.19	0.43	0.01	447	58	35
	**P46638**	**Rab11b**	**0.31**	**0.09**	**0.04**	**146**	**65**	**34**
	A2AG50	Map7d2	0.49	0.07	0.03	63	15	29
	P62717	Rpl18a	0.53	0.01	0.03	270	64	34
	Q9D7S7	Rpl22l1	0.56	0.03	0.04	66	46	32
	Q64455	Ptprj	0.81	0.00	0.05	16	9	29
	Q8CIF6	Sidt2	23.11	0.00	0.03	9	7	27
	Q3UHF7	Hivep2	24.09	0.00	0.00	9	1	28
	Q8BYI8	Kiaa1467	24.64	0.00	0.03	23	17	29
PSD	**Q9Z268**	**Rasal1**	**-24.90**	**0.00**	**0.01**	**106**	**37**	**31**
	P67871	Csnk2b	-1.19	0.00	0.05	52	62	31
	Q9WV69	Dmtn	-0.69	0.06	0.03	143	60	32
	P62137	Ppp1ca	-0.68	0.03	0.02	473	66	34
	Q99MK8	Adrbk1	-0.62	0.09	0.01	188	48	32
	P11627	L1cam	-0.60	0.06	0.03	781	39	36
	P46735	Myo1b	-0.45	0.19	0.05	134	27	32
	Q9WV92	Epb41l3	-0.44	0.14	0.03	666	60	34
	P47757	Capzb	-0.42	0.16	0.01	383	68	34
	P47754	Capza2	-0.40	0.17	0.04	471	86	34
	P47753	Capza1	-0.39	0.24	0.04	134	76	31
	Q62261	Sptbn1	-0.30	0.27	0.04	4844	82	38
	P16330	Cnp	0.36	0.72	0.03	311	61	33
	Q91VE0	Slc27a4	0.37	0.71	0.02	173	42	32
	P61264	Stx1b	0.48	0.46	0.03	521	63	35
	P58281	Opa1	0.61	0.31	0.03	926	67	35
	**O35393**	**Efnb3**	**1.08**	**0.09**	**0.05**	**98**	**48**	**31**
	Q64521	Gpd2	1.14	0.04	0.02	822	74	36
	Q80XI4	Pip4k2b	1.50	0.02	0.05	97	41	31
	**Q9CZT8**	**Rab3b**	**1.60**	**0.01**	**0.05**	**66**	**54**	**31**
	Q8BTH8	Csnk1g1	1.65	0.06	0.05	36	20	30
	O54983	Crym	22.38	0.00	0.01	170	50	33
	Q8C190	Vps9d1	22.74	0.00	0.01	9	7	29
	Q8BTY2	Slc4a7	23.72	0.00	0.02	55	14	30
	P24270	Cat	23.84	0.00	0.02	347	60	34
	P70195	Psmb7	24.04	0.00	0.02	80	52	32
	Q9R1P0	Psma4	24.08	0.00	0.00	72	46	32
	P50247	Ahcy	24.29	0.00	0.01	101	55	32
	P10649	Gstm1	24.43	0.00	0.02	146	65	33

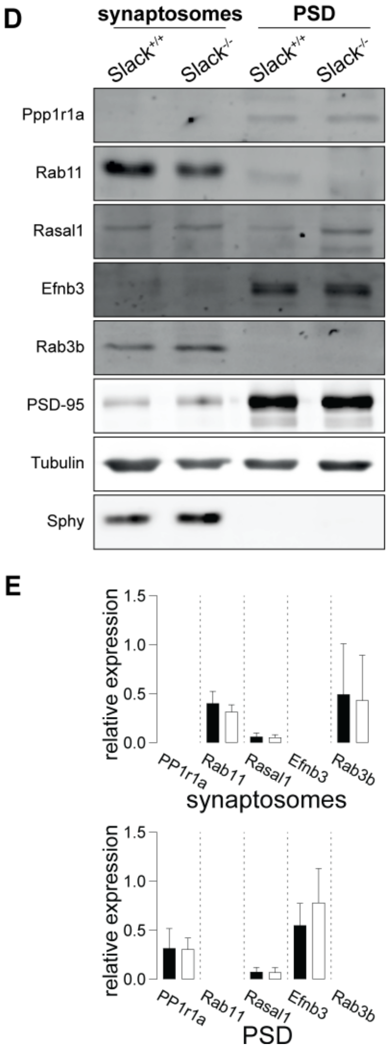

Figure S3. Synaptosomal and postsynaptic proteome of infant *Slack*$^{+/+}$ and *Slack*$^{-/-}$.

(**A**) Representative immunoblots of biochemical fractions from P9 *Slack*$^{+/+}$ and *Slack*$^{-/-}$ forebrains. Synaptosomal and postsynaptic density (PSD) enriched fractions were loaded and probed for activity regulated cytoskeleton associated protein (Arc) and fragile X mental retardation 1 (FMRP).

As control for biochemical fractionation, PSD-95 is enriched in PSD and de-enriched in synaptosomes. Pre-synaptic synaptophysin (Sphy) is absent from PSD and enriched in synaptosomes.

(**B**) Bar diagrams depicting Arc (n = 5) and FMRP (n = 5) protein band intensities normalized to α-tubulin in synaptosomal (left) and PSD (right) enriched fractions. Note that Arc was not detected in synaptosomal enriched fractions. Statistical significance was tested by paired t-test.

(**C**) Synaptosomal (n = 7 each for *Slack*$^{+/+}$ and *Slack*$^{-/-}$) and PSD (*Slack*$^{+/+}$, n = 8; *Slack*$^{-/-}$, n = 6) enriched fractions from biochemical fractionations of P9 *Slack*$^{+/+}$ and *Slack*$^{-/-}$ forebrains were analyzed using LC/MS-MS. Proteins with a significant alteration in one of the comparisons are sorted by *Slack*$^{-/-}$ over *Slack*$^{+/+}$ ratio for synaptosomal (top) and PSD enriched (bottom) fractions. Negative ratio indicates down-, positive upregulated proteins. Expression of bold-type proteins was further characterized by Western-blot in D.

(**D**) Representative immunoblots of biochemical fractions from P9 *Slack*$^{+/+}$ and *Slack*$^{-/-}$ forebrains. Synaptosomal and postsynaptic density (PSD) enriched fractions were loaded and probed for. As control for biochemical fractionation, PSD-95 is enriched in PSD and de-enriched in synaptosomes. Pre-synaptic synaptophysin (Sphy) is absent from PSD and enriched in synaptosomes.

(**E**) Bar diagrams depicting Ppp1r1a (n = 3), Rab11 (n = 5), Rasal1 (n = 3), Efnb3 (n = 4), and Rab3b (n = 5) protein band intensities normalized to α-tubulin in synaptosomal (left) and PSD (right) enriched fractions. Note that PP1r1a and Efnb3 were not detected in synaptosomal, Rab11 and Rab3b not in PSD enriched fractions. Statistical significance was tested by paired t-test.

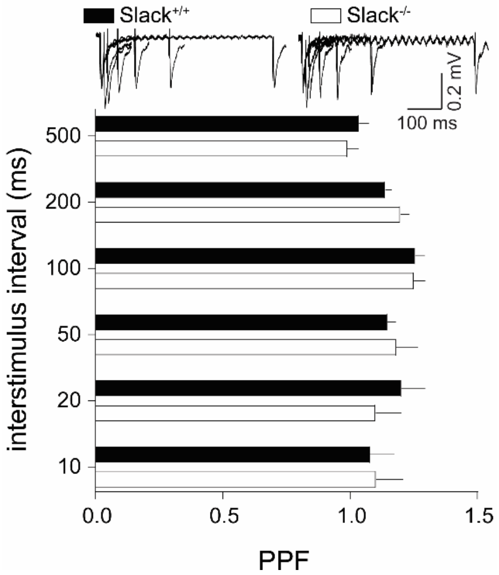

Figure S4. Normal presynaptic function in adult *Slack*$^{-/-}$.

Paired-pulse facilitation (PPF) of Schaffer-collateral fEPSP recorded from acute forebrain slices of P8 to P12 *Slack*$^{+/+}$ and *Slack*$^{-/-}$ mice was not different between *Slack*$^{+/+}$ (n = 15 slices from 8 animals) and *Slack*$^{-/-}$ (n = 10 slices from 3 animals) for all inter-stimulus intervals. Traces from representative recordings shown on top (*Slack*$^{+/+}$: 10 ms: 1.1 ± 0.10 mV, 20 ms: 1.2 ± 0.09 mV, 50 ms: 1.1 ± 0.03 mV, 100 ms: 1.3 ± 0.04 mV, 200 ms: 1.1 ± 0.03 mV, 500 ms: 1.0 ± 0.04 mV; *Slack*$^{-/-}$: 10 ms: 1.1 ± 0.11 mV, 20 ms: 1.1 ± 0.10 mV, 50 ms: 1.2 ± 0.09 mV, 100 ms: 1.3 ± 0.04 mV, 200 ms: 1.2 ± 0.04 mV, 500 ms: 1.0 ± 0.4 mV).

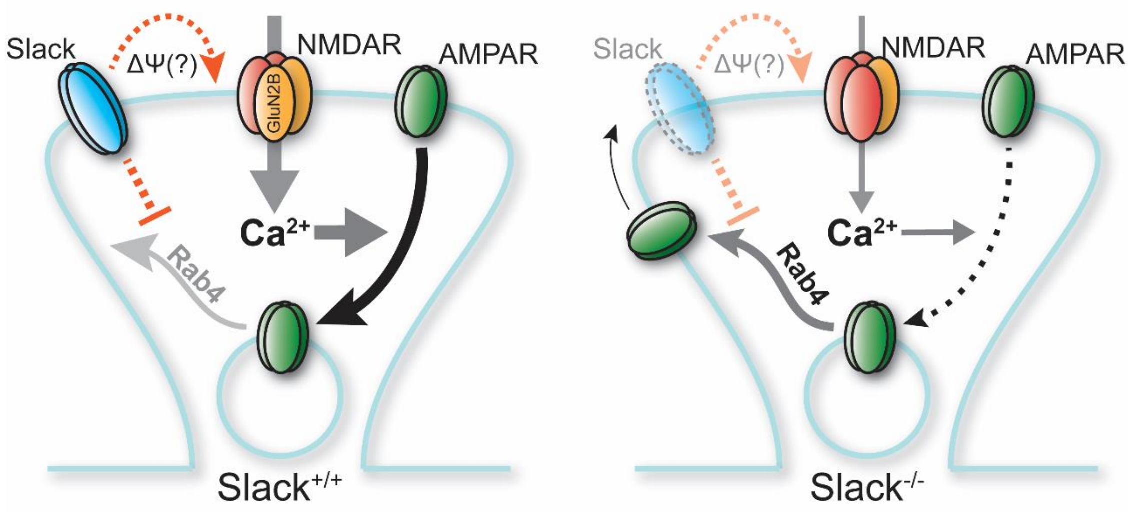

Figure S5. Model of Slack influencing hippocampal synaptic plasticity in neonates.

In wildtype *Slack*$^{+/+}$ mice (left panel), Slack promotes NMDAR-meditated AMPAR endocytosis by an unknown mechanism (pointed red arrow) which might be related to membrane repolarization, while suppressing Rab4-supported rapid recycling of AMPAR (red blocking arrow). In *Slack*$^{-/-}$, however, lacking amplification of NMDAR function prevents effective AMPAR endocytosis while increased Rab4 function might accelerate rapid recycling of newly-endocytosed AMPAR back to the plasma membrane, from where they can again enter the PSD. Additionally, NMDAR in infant *Slack*$^{-/-}$ contain less GluN2B than in *Slack*$^{+/+}$.
